# Chemical suppressors of *mlo-*mediated powdery mildew resistance

**DOI:** 10.1042/BSR20171389

**Published:** 2017-12-12

**Authors:** Hongpo Wu, Mark Kwaaitaal, Roxana Strugala, Ulrich Schaffrath, Paweł Bednarek, Ralph Panstruga

**Affiliations:** 1 Unit of Plant Molecular Cell Biology, Institute for Biology I, RWTH Aachen University, Worringerweg 1, 52056 Aachen, Germany; 2 Institute for Biology III, RWTH Aachen University, Worringerweg 1, 52056 Aachen, Germany; 3 Institute of Bioorganic Chemistry, Polish Academy of Sciences, 61-704 Poznán, Poland

**Keywords:** Arabidopsis thaliana, barley, chemical inhibitor screen, mlo resistance

## Abstract

Loss-of-function of barley mildew locus o (*Mlo*) confers durable broad-spectrum penetration resistance to the barley powdery mildew pathogen, *Blumeria graminis* f. sp. *hordei* (*Bgh*). Given the importance of *mlo* mutants in agriculture, surprisingly few molecular components have been identified to be required for this type of resistance in barley. With the aim to identify novel cellular factors contributing to *mlo*-based resistance, we devised a pharmacological inhibitor screen. Of the 41 rationally chosen compounds tested, five caused a partial suppression of *mlo* resistance in barley, indicated by increased levels of *Bgh* host cell entry. These chemicals comprise brefeldin A (BFA), 2′,3′-dideoxyadenosine (DDA), 2-deoxy-d-glucose, spermidine, and 1-aminobenzotriazole. Further inhibitor analysis corroborated a key role for both anterograde and retrograde endomembrane trafficking in *mlo* resistance. In addition, all four ribonucleosides, some ribonucleoside derivatives, two of the five nucleobases (guanine and uracil), some guanine derivatives as well as various polyamines partially suppress *mlo* resistance in barley via yet unknown mechanisms. Most of the chemicals identified to be effective in partially relieving *mlo* resistance in barley also to some extent compromised powdery mildew resistance in an *Arabidopsis mlo2 mlo6* double mutant. In summary, our study identified novel suppressors of *mlo* resistance that may serve as valuable probes to unravel further the molecular processes underlying this unusual type of disease resistance.

## Introduction

Powdery mildew is a common and widespread disease in angiosperm plants [1,2]. It is caused by obligate biotrophic fungal pathogens of the order Erysiphales (Ascomycetes; [3]). Barley (*Hordeum vulgare*) and *Arabidopsis thaliana* are monocotyledonous and dicotyledonous host plant species, respectively, for which the interaction with powdery mildew fungi has been studied extensively at the genetic, molecular, and cellular level [4–6]. In both instances, various plant immune pathways can limit the extent of fungal invasion. Recessively inherited loss-of-function alleles of mildew locus o (*Mlo*) genes confer a prominent type of highly effective powdery mildew resistance [7]*.* For example, barley *mlo* mutants exhibit non-race specific and durable resistance to virtually all isolates of the barley powdery mildew pathogen, *Blumeria graminis* f. sp. *hordei* (*Bgh*) [8–10]. This type of resistance, which has been widely deployed in European agriculture, is characterized by early cessation of host cell penetration at attempted infection sites [11]. The barley *Mlo* gene encodes for a member of an evolutionary conserved type of integral membrane protein with yet unknown biochemical activity [12,13].

To date, the molecular mechanisms underlying *mlo* resistance remain poorly understood. A few components have been identified to be required for *mlo*-mediated resistance in barley. Some of these were discovered in a forward genetic screen for suppressor mutants of *mlo* resistance, which led to the identification of partially susceptible individuals. These double mutants were subsequently found to be defective at two unlinked genetic loci termed *Required for mlo-specified resistance 1* (*Ror1*) and *Ror2* [14]. While the *Ror1* gene has not been cloned yet, *Ror2* encodes a member of the SNARE (Soluble *N*-ethylmaleimide-sensitive factor Attachment protein REceptor) superfamily [15]. Additional factors necessary for *mlo*-based resistance were detected by chemical inhibitor studies. For example, treatment of barley coleoptiles with 2-deoxy-d-glucose (interfering with callose biosynthesis) or exogenous application of calcium ions resulted in elevated levels of host cell entry [16,17]. Similarly, treatment with the actin depolymerization inhibitor cytochalasin E resulted in increased penetration by *Bgh* on barley *mlo* leaves [18]. Finally, transient overexpression of particular genes in single epidermal cells can (partially) overcome *mlo* resistance in barley. Examples comprise the ectopic expression of barley BAX inhibitor [19], certain actin-depolymerizing factors (ADFs; [18]), some calcium-dependent protein kinases (CDPKs; [20]), and dominant-negative forms of particular SNARE proteins [15,21].

Similar to barley, in *Arabidopsis* loss-of-function *mlo* mutants condition broad-spectrum powdery mildew resistance. However, *Arabidopsis mlo2* single mutants show only partial resistance, whereas full resistance requires a *mlo2 mlo6 mlo12* triple mutant [22]. The genetic resources available for *Arabidopsis* allowed dissecting the genetic requirements for *mlo* resistance at a broader scale than in barley. These efforts led to the insight that components of non-host resistance such as genes *PENETRATION1* (*PEN1*), *PEN2*, and *PEN3* as well as *CYP79B2/CYP79B3* also contribute to *mlo2*-conditioned partial resistance in *Arabidopsis* [22,23]. However, these genes are dispensable, alone or in combination, for complete immunity in the *mlo2 mlo6 mlo12* triple mutant [24]. Notably, PEN1 is the *Arabidopsis* ortholog of the t-SNARE Ror2, which is required for full *mlo* resistance in barley [15].

In the present study, we performed a chemical screen to identify novel components required for *mlo* resistance in barley. We focussed on a set of rationally selected substances that target a broad range of cellular activities. Of the five compounds found in the initial screen to increase *Bgh* entry rates in a *mlo* genotype, three were investigated in more detail. The results of our experiments further support a role for vesicle trafficking in *mlo* resistance and unravel polyamines as well as ribonucleosides, some of their derivatives and two of the four nucleobases as novel suppressors of *mlo* resistance in barley.

## Results

### A screen for chemical suppressors of *mlo*-mediated resistance in barley identifies five compounds that result in increased *Bgh* host cell entry

With the aim of identifying additional components and/or pathways involved in *mlo*-mediated powdery mildew resistance, we selected 41 chemicals (Supplementary Table S1) that are known to inhibit key cellular pathways or to regulate physiological processes involved in plant defense and tested them for their impact on the *Bgh* infection phenotype in barley *mlo* mutant plants. Due to the established link of Mlo protein function/*mlo* resistance to calcium signaling [7,20,25], the selected compounds covered a range of calcium transport/signaling inhibitors. Next to a solvent negative control, we applied each chemical at three different concentrations to leaves of a barley *mlo* null mutant via vacuum infiltration after removal of the abaxial leaf epidermis (see details in ‘Materials and methods’ section). Subsequently, the treated leaves were inoculated on the adaxial side with *Bgh* conidiospores and infection success was scored microscopically at 48 h post inoculation (hpi). We first validated this procedure by treatment with the known suppressor of *mlo* resistance, the actin polymerization inhibitor cytochalasin E (Supplementary Table S1). Consistent with results from a previous study [18], application of 5 µg/ml cytochalasin E resulted in a significant increase in host cell penetration by *Bgh* (9%) compared with the respective DMSO control (1%; Supplementary Figure S1).

The initial chemical screen based on 41 carefully selected compounds identified five substances that are each effective in partially suppressing *mlo* resistance in barley by increasing the *Bgh* penetration rate from ~1% in the respective solvent control to ~30% (Table 1 and Supplementary Figure S2). These chemicals include an inhibitor that blocks endosomal trafficking (brefeldin A (BFA)), a substance that interferes with cyclic adenosine monophosphate (cAMP) biosynthesis (2′,3′-dideoxyadenosine (DDA)), a compound that prevents callose formation (2-deoxy-d-glucose), a polyamine that is considered as positive regulator of plant immunity (spermidine), and a cytochrome P450 monooxygenase inhibitor that impedes biosynthesis of various antimicrobial compounds (1-aminobenzotriazole).

**Table 1:** Chemicals identified in the initial pharmacological screen that partially suppress *mlo* resistance in barley

Chemical	Described inhibitory effect	Most effective concentration (mM)^a^	*Bgh* host cell entry rate^b^
1-aminobenzotriazole	Cytochrome P450 monooxygenase inhibitor	0.05	5%
BFA	Vesicle trafficking inhibitor; interferes with protein secretion in eukaryotic cells	0.01	9%
2-deoxy-d-glucose	Callose biosynthesis inhibitor	1	8%
DDA	Adenylate cyclase inhibitor	0.2	14%
Spermidine	Triamine with unknown cellular target; known to promote plant immunity	2.5	32%

a Most effective concentration is the tested concentration at which the compound caused the highest *Bgh* entry rate. Solvent control entry rates were ~1% for all the chemicals (see also Figures 1–5 below).

b *Bgh* host cell entry was microscopically assessed as the proportion of germinated conidia that succeeded in the formation of microcolonies.

Based on the chemicals recognized as effective suppressors in the initial screen, we extended our analysis by testing additional compounds that are either known to have a similar inhibitory activity as the originally identified chemicals or that represent structural analogs of these substances (Supplementary Table S2). This follow-up analysis was meant to independently validate the results by functionally and/or structurally related compounds and to further narrow down potential cellular targets that are critical for *mlo*-mediated resistance in barley. We focussed in the following on three of the five identified chemicals, namely BFA, spermidine, and DDA. To gain insight whether these substances also affect *mlo* resistance in other plant species, we additionally explored the effect of these compounds on the interaction of a powdery mildew pathogen with the *Arabidopsis mlo2 mlo6* double mutant.

### Endosomal trafficking is required for *mlo*-mediated powdery mildew resistance

Endosomal trafficking plays an essential role in plant defense by relocalization of cellular structures and molecules to pathogen infection sites [26]. One well-known and widely used inhibitor of this process, BFA, which blocks exocytosis [27], was tested in the initial screen. Treatment with BFA (10 µM) increased the penetration success of *Bgh* in the barley *mlo* mutant to ~9% compared with ~1% in the DMSO solvent control (Table 1 and Figure 1A). To validate the effect of BFA, we tested another well-characterized exocytosis inhibitor, wortmannin [28–30]. Wortmannin treatment (20 µM) led to an increase in the *Bgh* penetration rate to ~8% in comparison with ~2% in the DMSO solvent control (Figure 1B). Based on the findings obtained with BFA and wortmannin, we tested three additional compounds with known impact on endocytosis, the dynamin inhibitors myristyltrimethylammonium bromide (MiTMAB; [31,32]) and dynasore [33] as well as the monovalent ion-selective ionophore monensin sodium [34]. Similar to the results obtained with BFA and wortmannin, all three tested substances were able to increase the penetration success of *Bgh* in the barley *mlo* mutant. The corresponding entry rates were ~14% for MiTMAB (500 µM; Figure 1C), ~9% for dynasore (100 µM; Figure 1D), and ~25% for monensin (2 µM; Figure 1E), each compared with ~1% in the respective solvent controls.

**Figure 1:**
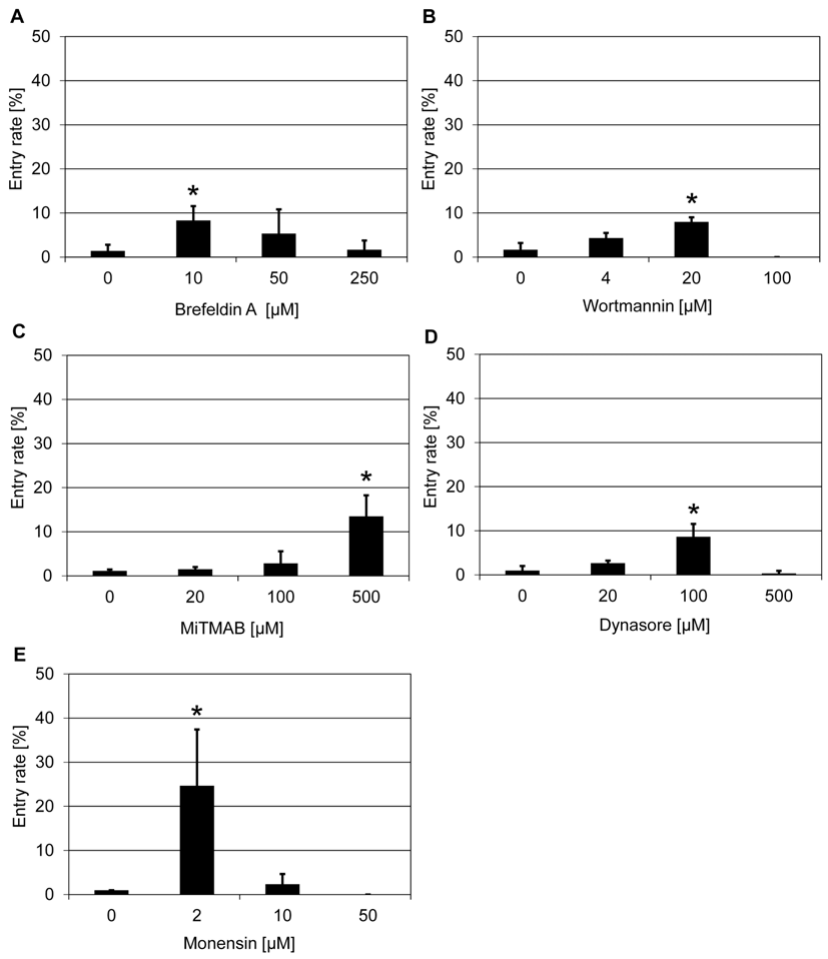
Pharmacological interference with endosomal trafficking increases penetration success of *Bgh* on a barley *mlo* mutant Excised leaves of the barley *mlo*-3 mutant were vacuum infiltrated with various concentrations of BFA (**A**), wortmannin (**B**), MiTMAB (**C**), dynasore (**D**), and monensin (**E**) as described in the ‘Materials and methods’ section. Subsequently, barley leaves were inoculated with *Bgh* conidiospores (isolate K1). Powdery mildew entry rates were microscopically evaluated at 48 hpi. Data shown represent the mean ± S.D. from three independent experiments, based on four leaves for each concentration in each experiment and >100 interaction sites evaluated per leaf (i.e. >1200 interaction sites per concentration in total). Asterisks above the columns indicate statistically significant differences (*P*<0.05; ANOVA one-way test; Bonferroni–Holm *post-hoc* analysis) compared with the respective solvent control (0 µM).

Given the findings in monocotyledonous barley, we also explored whether these chemicals affect *mlo* resistance in the dicotyledonous model plant *A. thaliana*. To this end, we focussed on the *mlo2 mlo6* double mutant, which provides a robust yet incomplete level of powdery mildew resistance [22]. Similar to the situation in barley, treatment with BFA (10 µM) increased the powdery mildew (*Golovinomyces orontii*) entry rate from ~5% (DMSO solvent control) to ~14% (Supplementary Figure S3A). Thus, BFA partially compromises *mlo*-based resistance in both monocotyledonous (barley) and dicotyledonous (*Arabidopsis*) plants. The impact of MiTMAB on resistance of the *Arabidopsis mlo2 mlo6* mutant was less clear; although at 500 µM the *G. orontii* entry rate was somewhat higher (~16%) compared with the solvent control (~10%), this difference was not statistically significant (Supplementary Figure S3B). Taken together, chemical inhibition supports the notion that endosomal trafficking is crucial for *mlo*-mediated resistance in barley and possibly also in *Arabidopsis*.

### Ribonucleosides, ribonucleoside derivatives, nucleobases, and nucleobase derivatives interfere with *mlo*-mediated powdery mildew resistance

**Figure 2:**
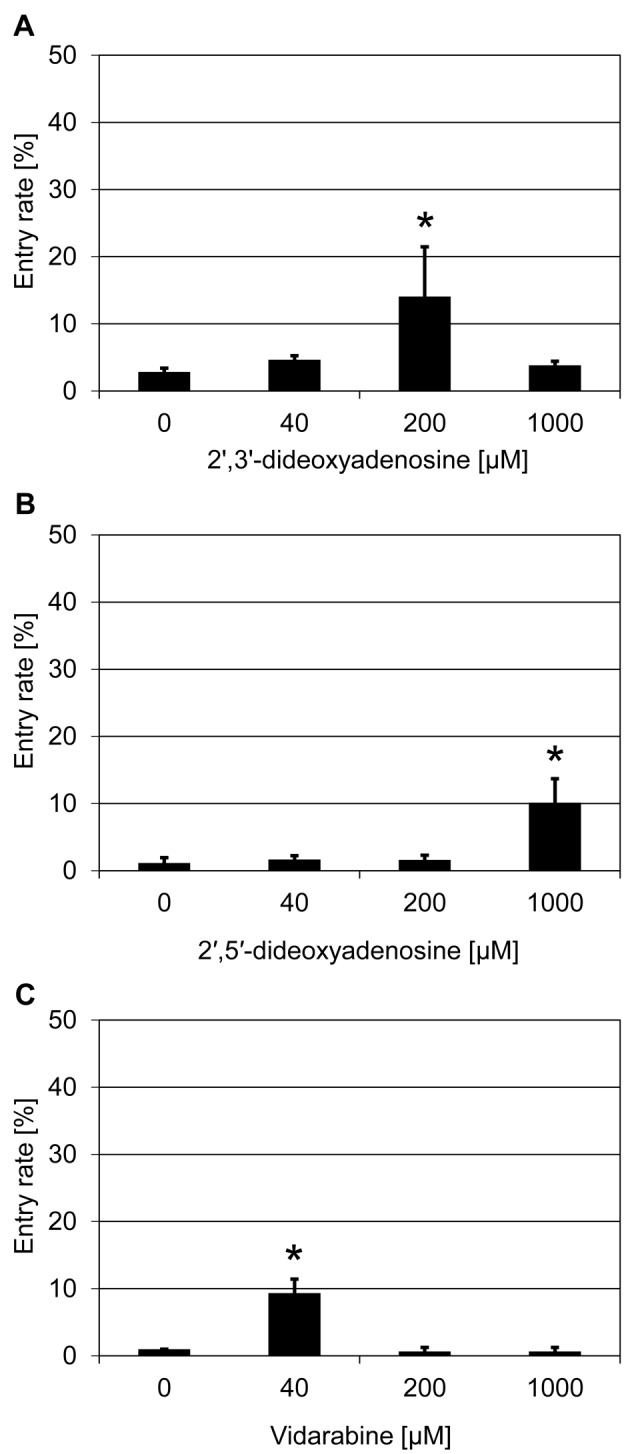
Nucleoside-derived adenylate cyclase inhibitors increase penetration success of *Bgh* on a barley *mlo* mutant Excised leaves of the barley *mlo-*3 mutant were vacuum infiltrated with various concentrations of DDA (**A**), 2′,5′-dideoxyadenosine (**B**), and vidarabine (**C**) as described in the ‘Materials and methods’ section. Subsequently, barley leaves were inoculated with *Bgh* conidiospores (isolate K1). Powdery mildew entry rates were microscopically evaluated at 48 hpi. Data shown represent the mean ± S.D. from three independent experiments, based on four leaves for each concentration in each experiment and >100 interaction sites evaluated per leaf (i.e. >1200 interaction sites per concentration in total). Asterisks above the columns indicate statistically significant differences (*P*<0.05; ANOVA one-way test; Bonferroni–Holm *post-hoc* analysis) compared with the respective solvent control (0 µM).

cAMP has been considered as a secondary messenger that bridges signal transduction between pathogen perception and downstream Ca^2+^ signaling in the signaling cascades of plant defense [35]. We initially tested one renowned inhibitor of this pathway, DDA, in our assay. This adenosine derivative is known to interfere with the activity of adenylate cyclase, the main enzyme responsible for catalyzing the generation of cAMP, and blocks cAMP signaling in plants [36]. Treatment with 200 µM DDA resulted in an increased penetration success of *Bgh* (~14%) in comparison with ~1% in case of the solvent control (Table 1 and Figure 2A). Subsequently, we tested two additional reported adenylate cyclase inhibitors, 2′,5′-dideoxyadenosine [37] and vidarabine (9-β-d-arabinofuranosyladenine; [38]), which are also adenosine derivatives. Both of them were effective in partially suppressing barley *mlo* resistance, with an increase in *Bgh* penetration to ~10% for 2′,5′-dideoxyadenosine treatment (1000 µM; Figure 2B) and to ~9% for vidarabine (40 µM; Figure 2C) compared with ~1% *Bgh* penetration success in the respective solvent controls.

Based on the finding that DDA, 2′,5′-dideoxyadenosine, and vidarabine are effective in partially suppressing *mlo* resistance in barley, we studied whether these inhibitors are also able to suppress partial *mlo* resistance in the *Arabidopsis mlo2 mlo6* double mutant. Treatment with DDA (200 µM) resulted in a statistically significant increase in penetration to ~25% as compared with ~8% in the solvent control (Supplementary Figure S4A). Similarly, application of 2′,5′-dideoxyadenosine (2000 µM) enhanced the *G. orontii* penetration rate to ~7% in comparison with ~2% in the solvent control (Supplementary Figure S4B). Treatment with vidarabine (200 µM) also caused an elevation in the host cell entry rate (~18%) as compared with ~9% in the solvent control; however, this increase was not statistically significant (Supplementary Figure S4C).

**Figure 3:**
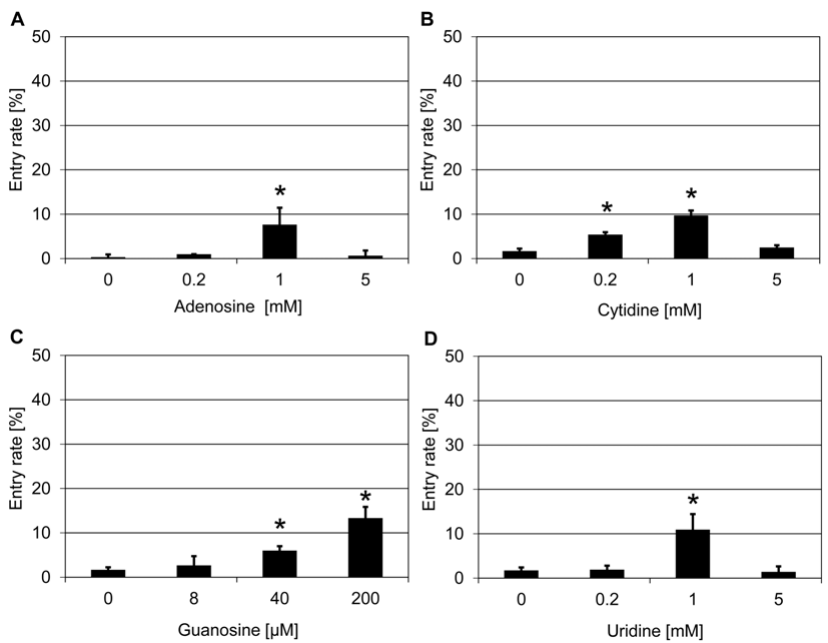
Ribonucleosides increase penetration success of *Bgh* on a barley *mlo* mutant Excised leaves of the barley *mlo-*3 mutant were vacuum infiltrated with three concentrations of adenosine (**A**), cytidine (**B**), guanosine (**C**), and uridine (**D**), respectively, as described in the ‘Materials and methods’ section. Subsequently, leaves were inoculated with *Bgh* conidiospores (isolate K1). Powdery mildew entry rates were microscopically evaluated at 48 hpi. Data shown represent the mean ± S.D. from three independent experiments, based on four leaves for each concentration in each experiment and >100 interaction sites evaluated per leaf (i.e. >1200 interaction sites per concentration in total). Asterisks above the columns indicate statistically significant differences (*P*<0.05; ANOVA one-way test; Bonferroni–Holm *post-hoc* analysis) compared with the respective solvent control (0 mM/µM).

To discriminate whether the partial suppression of *mlo* resistance by the tested chemicals is in fact due to an inhibition of cAMP biosynthesis or an effect related to other biochemical features of these nucleoside derivatives, we further analyzed the impact of non-modified ribonucleosides on *mlo* resistance in barley, which are expected not to interfere with cAMP signaling. Treatment with each of the four nucleosides (adenosine, cytidine, guanosine, and uridine) resulted in an increased penetration success by *Bgh*, yielding host cell entry rates of ~8–13% as compared with ~1–2% with the respective DMSO solvent controls (Figure 3). Of the four ribonucleosides, adenosine, cytidine, and uridine exerted their strongest effect at 1 mM, while guanosine was already very effective at 200 µM (Figure 3).

To investigate whether ribonucleosides are also effective in suppressing *mlo* resistance in *Arabidopsis*, we tested them in the context of the *Arabidopsis mlo2 mlo6* double mutant. Treatment with adenosine (0.2 mM) led to an increase in penetration success to ~5% from ~1% in the DMSO solvent control (Supplementary Figure S5A), although this increase was not statistically significant, while cytidine treatment did not change the penetration status with the concentrations tested ( Supplementary Figure S5B). Application of guanosine (5 mM) resulted in a significant increase in the entry rate to ~6% as compared with ~2% in the DMSO solvent control (Supplementary Figure S5C). Similarly, uridine treatment (25 mM) also significantly increased the entry rate of *G. orontii* to ~15% in comparison with ~1% in case of the DMSO solvent control (Supplementary Figure 5SD). Taken together, two of the four ribonucleosides (guanosine and uridine) were effective in partially suppressing *mlo* resistance in *Arabidopsis*.

**Figure 4:**
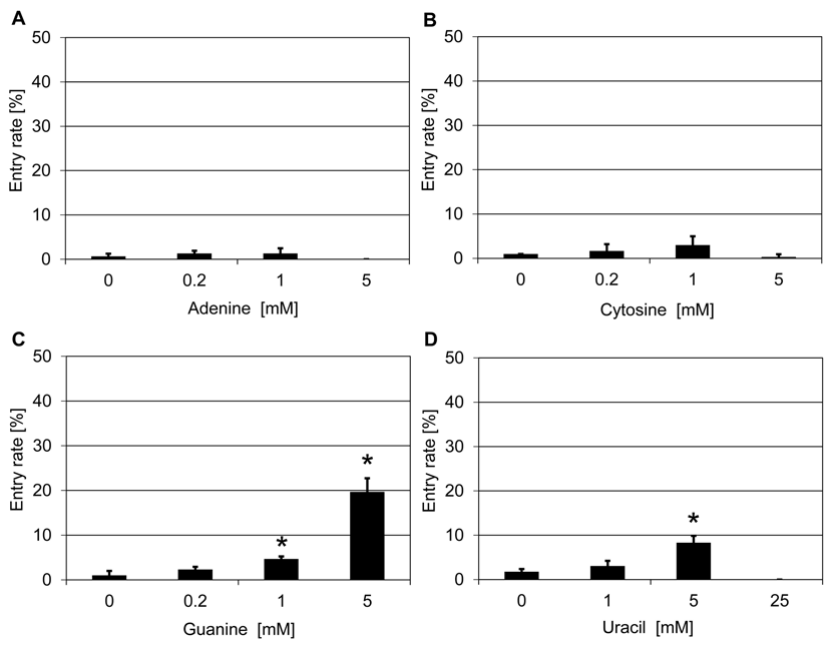
Nucleobases increase penetration success of *Bgh* on a barley *mlo* mutant Excised leaves of the barley *mlo-*3 mutant were vacuum infiltrated with three concentrations of adenine (**A**), cytosine (**B**), guanine (**C**), and uracil (**D**) as described in the ‘Materials and methods’ section. Subsequently, leaves were inoculated with *Bgh* conidiospores (isolate K1). Powdery mildew entry rates were microscopically evaluated at 48 hpi. Data shown represent the mean ± S.D. from three independent experiments, based on four leaves for each concentration in each experiment and >100 interaction sites evaluated per leaf (i.e. >1200 interaction sites per concentration in total). Asterisks above the columns indicate statistically significant differences (*P*<0.05; ANOVA one-way test; Bonferroni–Holm *post-hoc* analysis) compared with the respective solvent control (0 mM).

To further explore the structural requirements for the effectiveness of ribonucleosides and their derivatives, we tested four nucleobases (adenine, cytosine, guanine, and uridine), which are hydrolyzation products of the corresponding nucleosides, for their effect on *mlo* resistance in barley. Application of guanine (5 mM) and uracil (10 mM) significantly increased penetration success of *Bgh* to ~17–23% as compared with ~2% in the respective solvent controls (Figure 4C,D). By contrast, adenine and cytosine were not effective at the three concentrations tested (Figure 4A,B). Taken together, two nucleobases (guanine and uracil), four ribonucleosides (adenosine, cytidine, guanosine, and uridine) as well as the three tested ribonucleoside derivatives (DDA, 2′,5′-dideoxyadenosine, and vidarabine) were able to partially suppress *mlo* resistance in barley by increasing the *Bgh* host cell entry rate. Based on the result that guanine (5 mM) showed the strongest effect on the barley *mlo* mutant, we further tested guanine on the *Arabidopsis mlo2 mlo6* double mutant. This treatment (25 mM) increased the pentration success from ~2% in the water control to ~12% (Supplementary Figure S6).

**Figure 5:**
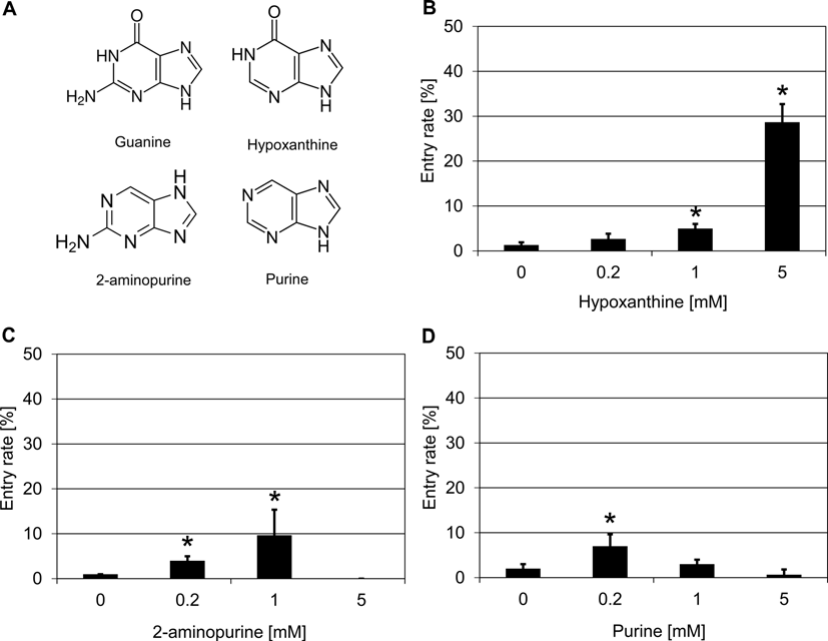
Guanine analogs increase penetration success of *Bgh* on a barley *mlo* mutant (**A**) Chemical structures of guanine and its analogs. (**B**–**D**) Treatment with hypoxanthine (B), 2-aminopurine (C), and purine (D) increase penetration success of *Bgh* on a barley *mlo* mutant. Excised leaves of the barley *mlo-*3 mutant were vacuum infiltrated with three concentrations of different chemicals as described in the ‘Materials and methods’ section. Subsequently, leaves were inoculated with *Bgh* conidiospores (isolate K1). Powdery mildew entry rates were microscopically evaluated at 48 hpi. Data shown represent the mean ± S.D. from three independent experiments, based on four leaves for each concentration in each experiment and >100 interaction sites evaluated per leaf (i.e. >1200 interaction sites per concentration in total). Asterisks above the columns indicate statistically significant differences (*P*<0.05; ANOVA one-way test; Bonferroni–Holm *post-hoc* analysis) compared with the respective solvent control (0 mM).

Given that guanine was effective in suppressing *mlo* resistance in both barley and *Arabidopsis*, we selected three analogs of guanine to investigate further the structural requirements for its mode of action. We focussed our activities on hypoxanthine (amino group at C2 position lacking), 2-aminopurine (oxygen at C6 position lacking) and purine (both the amino group at C2 position and the oxygen at C6 position lacking; Figure 5A). Treatment with hypoxanthine (5 mM) increased the *Bgh* penetration success to ~29% as compared with ~1% in the water solvent control (Figure 5B). Application of 2-aminopurine (1 mM) also led to an increase in the entry rate from ~1% in the water solvent control to ~10% (Figure 5C), while treatment with purine (0.2 mM) resulted in an increase in penetration success to ~7% compared with ~2% in the water solvent control (Figure 5D). Taken together, all three guanine analogs were effective in suppressing barley *mlo* resistance, though to various extents and at different concentrations.

### Polyamines interfere with *mlo*-mediated powdery mildew resistance

**Figure 6:**
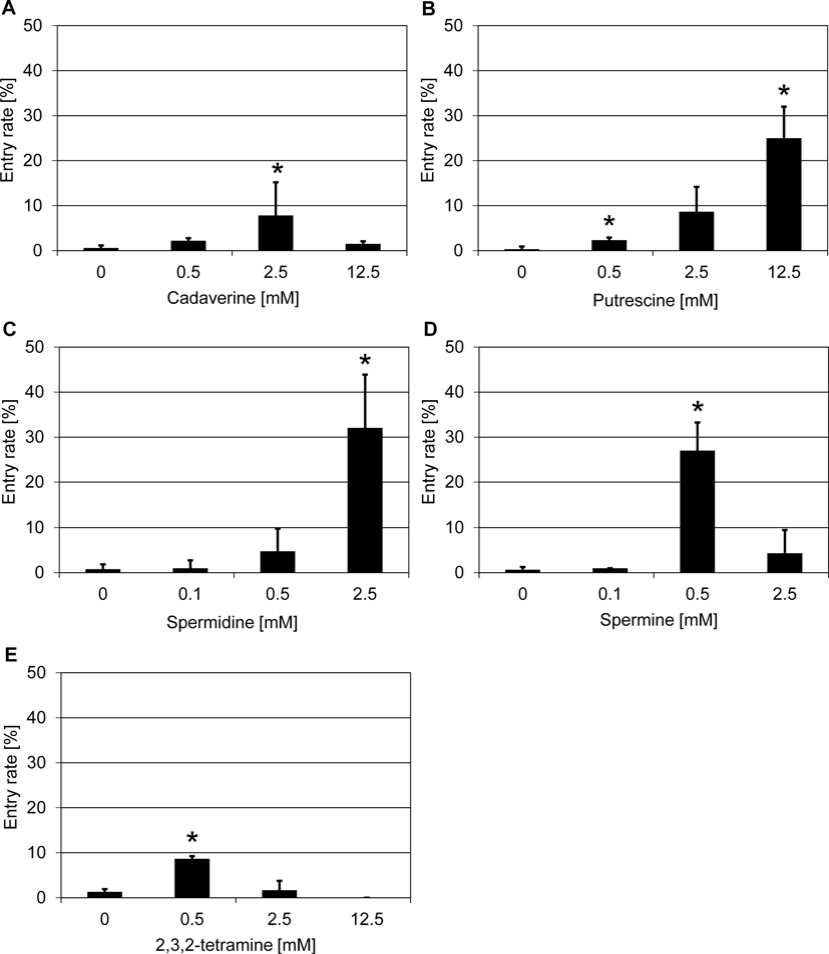
Polyamines increase penetration success of *Bgh* on a barley *mlo* mutant Excised leaves of the barley *mlo*-3 mutant were vacuum infiltrated with various concentrations of cadaverine (**A**), putrescine (**B**), spermidine (**C**), spermine (**D**), and 2,3,2-tetramine (**E**) as described in the ‘Materials and methods’ section. Subsequently, leaves were inoculated with *Bgh* conidiospores (isolate K1). Powdery mildew entry rates were microscopically evaluated at 48 hpi. Data shown represent the mean ± S.D. from three independent experiments, based on four leaves for each concentration in each experiment and >100 interaction sites evaluated per leaf (i.e. >1200 interaction sites per concentration in total). Asterisks above the columns indicate statistically significant differences (*P*<0.05; ANOVA one-way test; Bonferroni–Holm *post-hoc* analysis) compared with the respective solvent control (0 mM).

Polyamines are low molecular weight molecules involved in a number of biological processes, including gene expression, translation, cell proliferation, modulation of cell signaling, and membrane stabilization [39]. Polyamines were shown to act as positive regulators of plant defense to various phytopathogens, manifested by increased levels of these substances in leaves upon infection and a fungicide-like activity following their exogenous application [40–42]. In our initial screening, we first identified the triamine spermidine (Table 1), a polyamine species that also naturally occurs in barley [43]. Treatment with spermidine (2.5 mM) led to an increase in *Bgh* penetration success in the barley *mlo* mutant to ~32% as compared with ~1% in the water solvent control (Figure 6C). Subsequently, we tested more polyamine species, including putrescine and spermine, which are the precursor and conversion product, respectively, of spermidine, as well as cadaverine, 1,8-diamineoctane, 2,3,2-tetramine, and 1,12-diaminedodecane. These polyamines differ by the length of their carbon chains (four to twelve carbon atoms) and the number of amine groups (two to four), which under physiological conditions provide positive charges to this class of molecules (Table 2). Amongst the tested polyamines, cadaverine (2.5 mM) increased the *Bgh* penetration success to ~8% (Figure 6A), putrescine (12.5 mM) to ~25% (Figure 6B), 2,3,2-spermine (0.5 mM) to ~27% (Figure 6D), and 2,3,2-tetramine (0.5 mM) to ~10% (Figure 6E), respectively, compared with the solvent controls (all ~1%). Notably, exogenous application of the diamines 1,8-diamineoctane and 1,12-diaminedodecane did not change the penetration status of the barley *mlo* mutant at the three concentrations tested (Table 2 and Supplementary Figure S7). Taken together, the results of our experiments indicate that most of the polyamines tested are able to interfere with barley *mlo* resistance to *Bgh* by increasing the fungal penetration success (Table 2).

**Table 2:** Polyamine species tested in the pharmacological assay

Polyamine	Chemical formula	Chemical structure	Most effective concentration (mM)^a^	*Bgh* host cell entry rate (%)^b^
Putrescine	C_4_H_12_N_2_	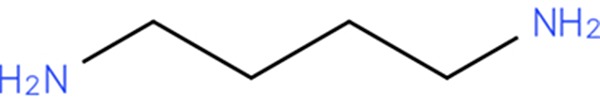	12.5	25
Cadaverine	C_5_H_14_N_2_	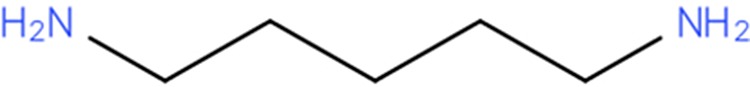	2.5	8
1,8-diamineoctane	C_8_H_20_N_2_	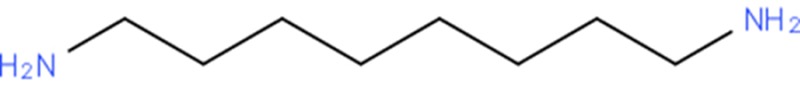	No effect	1
Spermidine	C_7_H_19_N_3_	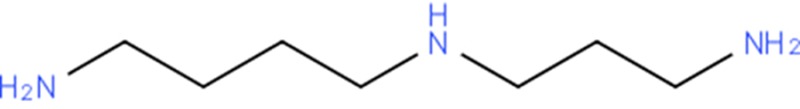	2.5	32
2,3,2-tetramine	C_7_H_20_N_4_	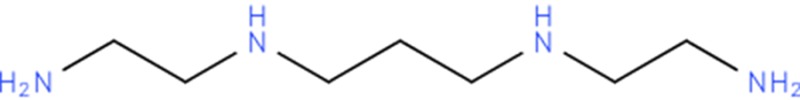	0.5	10
1,12-diaminedodecane	C_12_H_28_N_2_	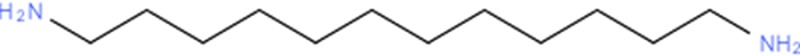	No effect	1
Spermine	C_10_H_26_N_4_	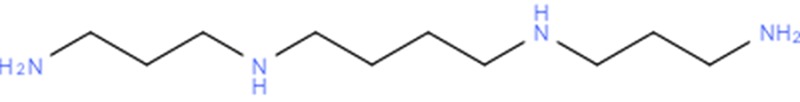	0.5	27

a Most effective concentration is the tested concentration at which the compound caused the highest *Bgh* entry rate.

b *Bgh* host cell entry was microscopically assessed as the proportion of germinated conidia that succeeded in the formation of microcolonies.

In order to investigate the activity spectrum of polyamine species further, we tested the most potent polyamine identified in the barley chemical screen (spermine) on the *Arabidopsis mlo2 mlo6* double mutant. In contrast with the barley *mlo* mutant (Figure 6D), spermine treatment did not change the penetration rate of *G. orontii* in the *mlo2 mlo6* double mutant at the three concentrations tested (Supplementary Figure S8).

### Endogenous polyamine levels do not differ between *Mlo* and *mlo* genotypes at early time points upon *Bgh* attack

Previously, increased endogenous levels of polyamines were found to correlate with resistance of barley to powdery mildew [40,44]. Since exogenous application of polyamines negatively affected resistance of the barley *mlo* mutant against *Bgh*, polyamines might be important substances modulating *mlo* resistance, perhaps via changes in their endogenous concentrations upon *Bgh* attack. To experimentally test this possibility, we measured the levels of key polyamines (putrescine, spermidine, and spermine) in leaves of both non-inoculated and inoculated (*Bgh* isolate K1) barley wild-type (*Mlo*) and *mlo* mutant plants in a time-course experiment at 0, 12, and 24 hpi via HPLC analysis. Results from this experiment revealed that the levels of putrescine increased in both inoculated and non-inoculated leaf samples at 12 and 24 hpi (~170–300 nmol/g fresh weight (FW)) compared with the 0 hpi time point (~60–70 nmol/g FW), possibly as a consequence of the experimental procedure (e.g. physical stress). Notably, the inoculated leaf samples showed lower putrescine levels (~170–200 nmol/g FW) than the non-inoculated leaf samples (~240–300 nmol/g FW), both for wild-type and *mlo* plants, with no statistically significant difference, neither between the two time points (12 and 24 hpi) nor between the two genotypes (*Mlo* and *mlo*; Supplementary Figure S9A). Spermidine levels transiently increased from ~80–100 nmol/g FW at 0 hpi to ~120 nmol/g FW at 12 hpi in inoculated leaf samples only, independent of the genotype, and declined to resting levels at 24 hpi (Supplementary Figure S9B). Spermine levels decreased significantly from ~40–50 nmol/g FW at 0 and 12 hpi to ~10–20 nmol/g FW at 24 hpi, irrespective of the inoculation status and genotype (Supplementary Figure S9C). In summary, we did not observe any marked genotype-specific alterations in polyamine levels upon inoculation with *Bgh* in our set of experiments.

### Polyamines act additively to the effect of barley *ror* mutants and impair *R* gene-mediated resistance and basal resistance to *Magnaporthe oryzae*

Since amongst the compounds identified in the original chemical screen, the tetramine spermidine showed the greatest effect on *Bgh* host cell entry in the barley *mlo* mutant (Table 1), we decided to further study the effect of polyamines on plant defense in more detail. In the following, we focussed on the tetramine spermine, which compared with spermidine showed a similar effect, yet at a five-fold lower concentration (0.5 mM compared with 2.5 mM; Table 2 and Figure 6C,D), potentially minimizing any side effects these types of compounds may have. We concentrated on the effect of spermine on suppressor mutants of *mlo* resistance, on *R* gene-mediated isolate-specific immunity and on the interaction of barley with the fungal rice blast pathogen, *Magnaporthe oryzae*, which is also modulated by *Mlo*.

**Figure 7:**
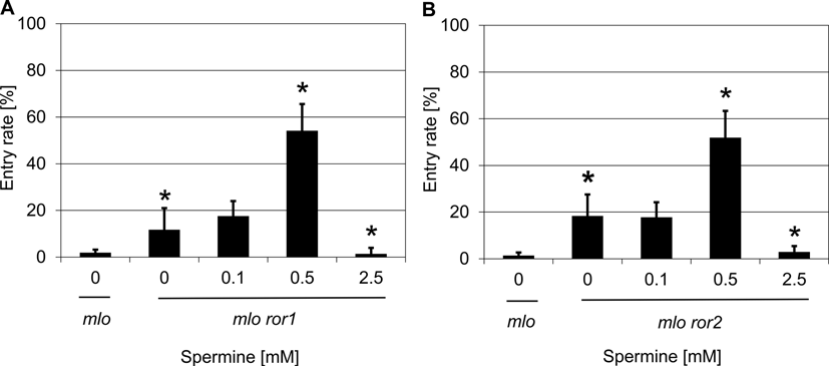
Spermine increases penetration success of *Bgh* on barley *mlo ror1* and *mlo ror2* double mutants Excised leaves of barley *mlo*-5 *ror1*-4 (**A**) and *mlo*-5 *ror2*-1 (**B**) double mutants were vacuum infiltrated with various concentrations of spermine as described in the ‘Materials and methods’ section. The barley *mlo*-5 mutant served as an additional control. Subsequently, leaves were inoculated with *Bgh* conidiospores (isolate K1). Powdery mildew entry rates were microscopically evaluated at 48 hpi. Data shown represent the mean ± S.D. from three independent experiments, based on four leaves for each concentration in each experiment and >100 interaction sites evaluated per leaf (i.e. >1200 interaction sites per concentration in total). Asterisks above the columns indicate statistically significant differences (*P*<0.05; ANOVA one-way test; Bonferroni–Holm *post-hoc* analysis) compared with the respective solvent control (0 mM).

To investigate whether polyamines exert their suppressive effect on *mlo* mutant plants through interfering with known components required for barley *mlo* resistance, we analyzed the barley *mlo ror1* and *mlo ror2* double mutants. Besides lacking a functional *Mlo* copy, these mutants are defective in *Ror* genes and show a partial relief in *mlo*-mediated resistance, as evidenced by enhanced susceptibility to *Bgh* [14]. Treatment with spermine (0.5 mM) significantly increased the *Bgh* penetration rate to ~54% as compared with ~12% in the water solvent control in case of the *mlo ror1* double mutant (Figure 7A). Likewise, spermine application (0.5 mM) also resulted in a significant increase in penetration success (~52% in comparison with ~18% in the water solvent control) in the *mlo ror2* double mutant (Figure 7B). Taken together, in addition to the suppressing effect of the *ror* mutants on *mlo*-mediated resistance, spermine was able to enhance susceptibility of barley *mlo ror* double mutants further. This additive effect on host cell entry suggests that spermine may interfere with a cellular pathway that is different from the ones affected in the *mlo ror* double mutants.

**Figure 8:**
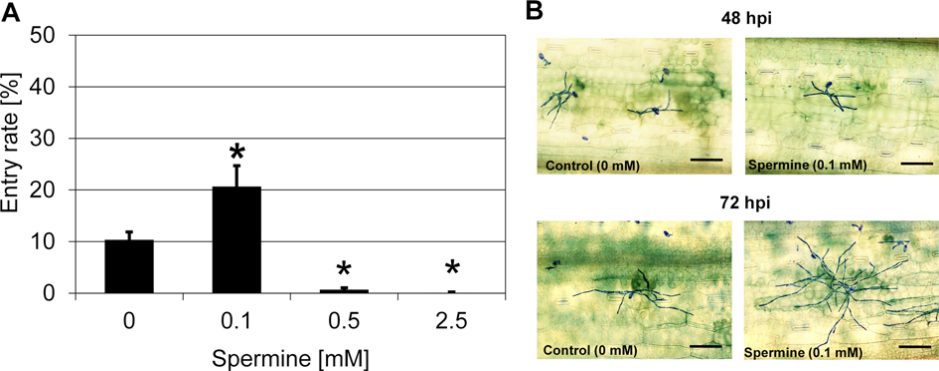
Spermine partially abolishes *Mla1*-mediated resistance to *Bgh* (**A**) Treatment of barely line P01 (*Mla1*) with spermine increases penetration success of *Bgh* (K1). Excised leaves of barley line P01 were vacuum infiltrated with various concentrations of spermine as described in the ‘Materials and methods’ section. Subsequently, leaves were inoculated with *Bgh* conidiospores (isolate K1). Powdery mildew entry rates were microscopically evaluated at 48 hpi. Data shown represent the mean ± S.D. from three independent experiments, based on four leaves for each concentration in each experiment and >100 interaction sites evaluated per leaf (i.e. >1200 interaction sites per concentration in total). Asterisks above the columns indicate statistically significant differences (*P*<0.05; ANOVA one-way test; Bonferroni–Holm *post-hoc* analysis) compared with the solvent control (0 mM). (**B**) Cell death suppression effect of spermine. Leaves of barley line P01 (*Mla1* genotype) were vacuum infiltrated with water or 0.1 mM spermine and then inoculated with *Bgh* conidiospores (isolate K1). At 48 hpi (upper panel) or 72 hpi (lower level) after treatment with water (left) or 0.1 mM spermine (right), leaves were stained with Trypan Blue as described in ‘ Materials and methods ’ section. Micrographs shown are representatives for the results of two independent experiments. Scale bar: 100 µm.

To examine whether polyamines have a similar effect on other types of plant resistance, we tested spermine in the interaction between barley line P01, harboring the isolate-specific mildew locus a (*Mla1*) immune receptor gene, and *Bgh* isolate K1, which carries the cognate *AvrMla1* avirulence gene. The interaction between these two partners is characterized by a low host cell entry rate, typically accompanied by a hypersensitive cell death response [6,45]. Treatment with spermine (0.1 mM) increased the penetration success of *Bgh* (isolate K1) on line P01 from ~10% in the solvent control to ~21% (Figure 8A). This enhanced entry rate, which is caused by a five times lower concentration than in the case of the barley *mlo* genotype (Figure 6D), correlates with a seeming decrease in the extent of host hypersensitive cell death and a concomitant increase in fungal microcolony size (Figure 8B).

**Figure 9:**
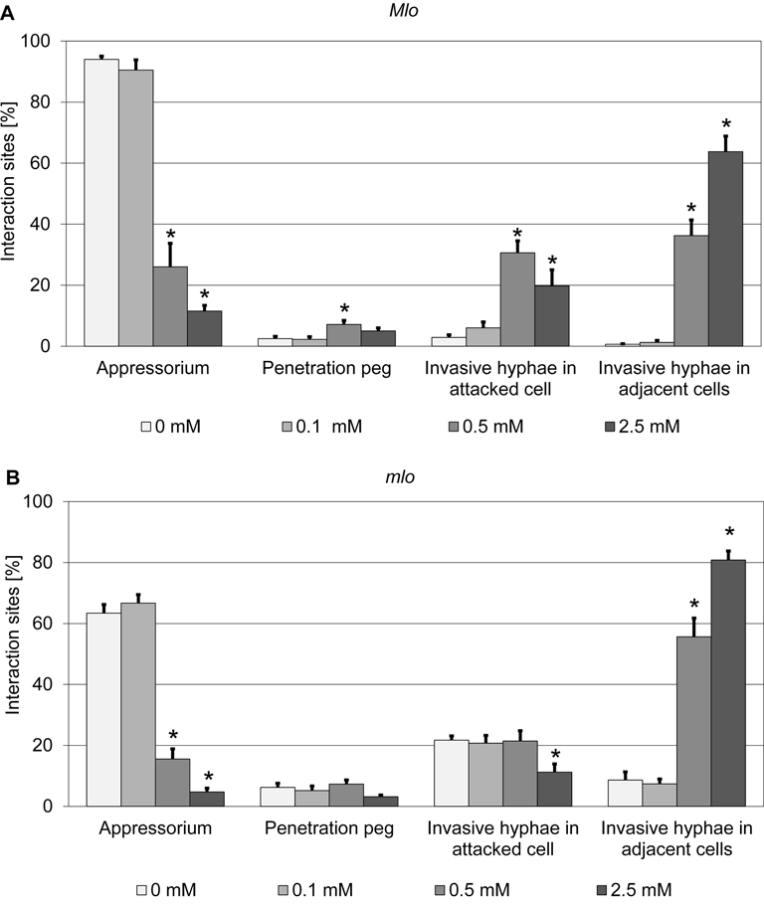
Spermine increases susceptibility of barley *Mlo* and *mlo* genotypes to *M. oryzae* Leaves of barley wild-type *Mlo* (**A**) and the *mlo*-3 mutant (**B**) were vacuum infiltrated with various concentrations of spermine as described in the ‘Materials and methods’ section and then spray inoculated with 200000 spores per ml of *M. oryzae* isolate TH6772. Microscopic evaluation of *M. oryzae* infection structures was performed at 48 hpi. More than 100 interaction sites per leaf and four leaves per concentration were assessed in each experiment. Interaction sites were assigned to four categories (‘appressorium’, ‘ penetration peg’, invasive hyphae in attacked cells’ and invasive hyphae in adjacent cells’). Data shown represent the mean ± S.D. For the wild-type (*Mlo*) genotype, results from two independent experiments were combined, and for the *mlo*-3 mutant, results from three independent experiments were combined. Asterisks above the columns indicate statistically significant differences (*P*<0.05; ANOVA one-way test; Bonferroni–Holm *post-hoc* analysis) compared with the respective solvent control (0 mM).

In contrast with the durable broad-spectrum resistance phenotype in response to *Bgh*, barley *mlo* mutants show enhanced susceptibility to the rice blast pathogen *M. oryzae* [46]. To investigate whether polyamines also interfere with the interaction of barley with *Magnaporthe*, we tested spermine by exogenous application on both barley wild-type (*Mlo*) and *mlo* mutant plants followed by challenge with *M. oryzae* isolate TH6772. In this set of experiments, both genotypes showed enhanced susceptibility to *M. oryzae* after treatment with 0.5 and 2.5 mM spermine. This phenotype was evidenced by an increase in the percentage of invasive fungal hyphae in attacked cells (*Mlo* genotype) and its adjacent cells (*Mlo* and *mlo* genotypes; Figure 9). In the wild-type (*Mlo* genotype), the number of invasive hyphae in the attacked cells increased from ~3% in the solvent control to ~31% (0.5 mM) and ~20% (2.5 mM). This effect was even more pronounced in the adjacent cells, where the percentage of invasive hyphae raised from ~1% (solvent control) to ~36% (0.5 mM) and ~64% (2.5 mM), respectively. In the case of the barley *mlo* mutant, the background level of invasive hyphae in the attacked (~22%) and adjacent cells (~9%) was higher in the control leaves (0 mM) than in the *Mlo* genotype. In contrast with the wild-type, the percentage of invasive hyphae remained unaltered (0.5 mM; ~21%) or decreased (2.5 mM; ~7%) in the attacked cells of the *mlo* genotype, while in the adjacent cells the increase was even stronger than in the *Mlo* genotype (~56% at 0.5 mM and ~81% at 2.5 mM). Taken together, spermine treatment promotes the formation of *M. oryzae* invasive hyphae in the adjacent cells of both *Mlo* and *mlo* genotypes, while this effect is restricted to the *Mlo* genotype with regard to the attacked cells.

## Discussion

In the present study, we followed a chemical approach to identify novel components/pathways required for *mlo*-based resistance. This work thus complements previous genetic analysis, which unraveled few genes required for *mlo*-mediated resistance in barley (*Ror1* and *Ror2*; [14,15]) and *Arabidopsis* (*PEN1, PEN2, PEN3, CYP79B2/CYP79B3*; [22,23]). In our initial chemical screen, we tested 41 compounds and identified five substances that partially restore *Bgh* host cell entry on a barley *mlo* mutant upon exogenous application via vacuum infiltration (Table 1). This seemingly high success rate might be because the 41 compounds were carefully chosen to target a broad range of cellular activities. Most of these substances are renowned inhibitors and have been reported to be effective in disrupting or regulating specific pathways and/or components in plant and/or animal cells. The concentrations of the chemicals used in our assay were devised based on publications and/or recommendations by the vendors. They were chosen such that with the three concentrations deployed in the experiments we could cover a comparatively broad spectrum (5-times dilution series; 25-times difference between lowest and highest concentration). Nonetheless, we cannot exclude the possibility that we missed substances that might suppress *mlo* resistance simply due to the limited concentration range tested. Additionally, potentially effective substances could be obscured by toxic effects on the fungal interaction partner, which might directly or indirectly get in contact with the respective compounds. Finally, stability of the chemicals in the apoplast and/or cytoplasm as well their membrane permeability are key parameters that may modulate their inhibitory activity. These effects, alone or in combination, may also account for the fact that for some chemicals an apparent dose-response relationship was seen (e.g. Figures 1B, 3B,C, and 4C), while in the case of other substances seemingly only single concentrations were effective (e.g. Figures 1C,E, 2B,C, and 3A,D). While it seems more plausible that these chemical inhibitors infiltrated into plant tissue negatively affect plant defense responses and thus compromise *mlo*-based resistance, we cannot completely rule out, as an alternative scenario, that the compounds exert a positive effect on fungal virulence.

Broad-spectrum resistance conferred by loss-of-function *mlo* genotypes has been reported to exhibit joint histological and phytopathological characteristics with plant non-host resistance. Accordingly, there are some shared genetic components that are required for both, *mlo* resistance and non-host resistance, suggesting a mechanistic overlap between the two types of plant defense [47]. This notion is reinforced by the fact that *Mlo* is co-expressed with components of non-host resistance, which altogether form an evolutionarily conserved regulon that is assumed to function as a module in plant immunity [48]. The fact that the polyamine spermine not only partially suppresses *mlo* resistance in barley (Figure 6), but also affects *Mla1*-conditioned *R* gene-mediated resistance (Figure 8) and defense against the rice blast pathogen *M. oryzae* (Figure 9) indicate that at least this compound is not a specific inhibitor of *mlo* resistance. Instead, it seems to affect a cellular pathway of basal defense critical for different types of plant immunity.

Of the five substances identified in the initial screen (BFA, DDA, 2-deoxy-d-glucose, spermidine, and 1-aminobenzotriazole; Table 1) we analyzed three in more detail. We refrained from further studying the callose biosynthesis inhibitor 2-dedoxy-d-glucose and the cytochrome P450 inhibitor 1-aminobenzotriazole since for these chemicals only limited choices of functional or structural analogs are commercially available. For the remaining three chemicals (BFA, DDA, and spermidine), we selected functional and/or structural analogs and additionally studied the effect of the original compound or its derivatives on the *Arabidopsis mlo2 mlo6* double mutant or other types of disease resistance.

BFA is a fungal metabolite that inhibits vesicle trafficking by targetting ADP-ribosylation factors (ARFs), small GTP-binding proteins that are involved in the transport of vesicles between the endoplasmic reticulum (ER) and the Golgi complex and within the Golgi complex itself [49]. BFA treatment of wild-type grapevine and *Arabidopsis* leaves lead to a partial suppression of penetration resistance to non-adapted powdery mildew pathogens, possibly by inhibiting transport of antifungal cargo (e.g. PEN1 SNARE protein, callose biosynthesis machinery) to attempted penetration sites [50]. Wortmannin is a specific inhibitor of phosphatidylinositol-3 (PI3) and phosphatidylinositol-4 (PI4) kinases. It interferes with protein sorting to the plant vacuole and causes formation of mixed compartments of *trans*-Golgi network and multivesicular bodies, which may lead to inhibition of exocytosis [29,30,51]. In our assay, BFA partially suppressed *mlo* resistance of both barley and *Arabidopsis* (Figure 1A and Supplementary Figure S3A), probably through inhibiting exocytosis. This notion was further corroborated by the results obtained with wortmannin (Figure 1B), indicating that exocytosis might be critical for *mlo*-mediated resistance. MiTMAB is a dynamin GTPase inhibitor, which acts by interfering with binding of phospholipids to the pleckstrin homology (PH) domain of dynamin [32]. Application of MiTMAB blocks multiple forms of endocytosis in animal cells [32,52]. Dynasore is an GTPase inhibitor of dynamin 1 and dynamin 2, which acts as an inhibitor of dynamin-dependent endocytosis by blocking coated vesicle formation [33]. Monensin acts as a monovalent ionophore that inhibits receptor-dependent internalization of some proteins and perturbs the structure of the Golgi apparatus by disrupting transmembrane ion gradients [34,53,54]. However, up to now, the inhibitory roles of MiTMAB and dynasore have been experimentally proven only in animal cells; whether they exert the same activity in plant cells is still elusive. In our experiments, treatment with the three potential endocytosis inhibitors (MiTMAB, dynasore, and monensin) partially suppressed *mlo* penetration resistance of barley (Figure 1C–E). In addition, MiTMAB was also effective in moderately affecting *mlo* resistance in *Arabidopsis* (Supplementary Figure S3). These findings suggest that plant dynamins could be targets of MiTMAB and dynasore, and they suggest that dynamin-mediated endocytosis and ion gradient related internalization might be required for *mlo* resistance. Thus, both anterograde (exocytic) and retrograde (endocytic) vesicle transport seem to be necessary for *mlo* resistance in both monocot and dicot plants. This conclusion is consistent with previous findings that t-SNARE protein activity and actin cytoskeleton function are crucial for full *mlo* resistance in barley [15,18,21].

Ribonucleosides are hydrolyzation products of the main building blocks of RNA – the ribonucleotides. The latter form all types of cellular RNA, including mRNA, rRNA, tRNA, and miRNA. Nucleoside derivatives have been reported to inhibit viral replication in case of virus-infected cells, e.g. by restraining the activity of viral RNA-dependent RNA polymerase [55,56]. Additionally, in particular adenosine derivatives can also serve as inhibitors of cAMP biosynthesis/signaling [37,38]. However, the existence and physiological relevance of cAMP signaling in plants is still controversially discussed [35]. A partial suppressing activity on *mlo* resistance in barley was found with all four ribonucleosides, some ribonucleoside derivatives (DDA, 2′,5′-dideoxyadenosine, and vidarabine), two of the four nucleobases (guanine and uracil) and three nucleobase derivatives (hypoxanthine, 2-aminopurine, and purine, Figures 2–5). In addition two ribonucleosides (guanosine and uridine), some ribonucleoside derivatives (DDA and 2′,5′-dideoxyadenosine) and one of the four nucleobases (guanine) were also able to partially suppress *mlo* resistance in *Arabidopsis* (Supplementary Figure S4–S6). We excluded (ribo-)nucleotides from our experiments since these compounds are known to show poor cell permeability [57]. Although all effective substances exhibit some general relatedness at the functional level (all are hydrolyzation products of ribonucleotide monophosphates), chemically they can be broadly divided into two major groups – purine bases and their derivatives, and pyrimidine bases and their derivatives. We noted that generally members of both groups have the capacity to interfere with *mlo* resistance, which is a surprising finding given that purines and pyrimidines differ in their *de novo* biosynthetic, salvage, and catabolic pathways, which do not share common intermediates [58]. One possible explanation for this unexpected outcome is that the organic bases, which are a common feature of all these substances, impede particular cellular processes that rely on both types of bases, e.g. transcription and/or translation. Another possibility is the inhibition of cAMP signaling, which can be achieved by both purine and pyrimidine nucleotides. Bacterial and animal adenylyl cyclases, key enzymes catalyzing the biosynthesis of cAMP, are sensitive to inhibition by various nucleotides [59,60]. For this scenario to be true in our case, conversion of the exogenously applied nucleobases/nucleosides to nucleotides via salvage pathways has to be assumed. The fact that salvage is more effective for nucleosides, which required lower concentrations than nucleobases to interfere with *mlo* resistance (Figures 3 and 4), is principally supportive of this idea. Finally, nucleosides and their derivatives may exert a different type of inhibitory activity than nucleobases, and/or purines could act differently from pyrimidines. It will require additional work to unravel the exact mode of action by which these chemicals contribute to the partial suppression of *mlo* resistance.

Polyamines were previously considered as positive regulators of plant defense. Increased endogenous levels of polyamines were, for example found to correlate with resistance of barley to powdery mildew [40,44,44]. However, in contrast with this general belief, in our assay exogenous application of five polyamines partially suppressed *mlo* penetration resistance (Figure 6 and Table 2). In addition, spermine treatment partially abolished barley *Mla1-*mediated isolate-specific resistance to *Bgh* (Figure 8) and in part relieved barley basal resistance to the rice blast pathogen *M. oryzae* (Figure 9). Together these findings support the notion that apart from its stimulatory role, polyamines can also serve as negative modulators of plant defense. Precedence for such an inhibitory role of polyamines was found in the case of transgenic tomato lines hyperaccumulating spermidine; such lines were observed to be more susceptible to the necrotrophic fungal pathogen, *Botrytis cinerea* [61]. The presumed dual role of polyamines as positive or negative regulators could be dose dependent, with high levels negatively affecting plant defense, and with low levels supporting plant defense. Notably, many phytopathogenic fungi are capable of synthesizing polyamines, including spermine and spermidine [62]. This likely also applies to *Bgh*, whose genome encodes a spermine/spermidine synthase family protein (bgh03771; [63]). Polyamines may in fact play a role in fungal virulence, as experimental data suggest in the case of the maize – *Ustilago maydis* pathosystem [64].

Treatment with different concentrations of polyamines (12.5 mM putrescine, 2.5 mM spermidine, and 0.5 mM spermine) increased the penetration success of *Bgh* to similar levels (~25-30%; Figure 6B–D). Interestingly, the respective most effective concentration for the three polyamines exhibits a gradient pattern, which correlates with an increase in positive charges for each polyamine (di-, tri-, and tetramine, respectively; see Table 2). One may thus speculate that in particular the number of positive charges affiliated with polyamines, possibly chain length as well, determines their inhibitory effect on *mlo* resistance. This speculation is supported by the fact that diamines with a higher number of carbon atoms (such as 1,8-diamineoctane and 1,12-diaminododecane) failed to affect *mlo* resistance in barley at the concentrations tested (Table 2 and Supplementary Figure S7). However, we noted that treatment with 0.1 mM spermine was insufficient to suppress *mlo* resistance to *Bgh* (Figure 6D), basal resistance to *M. oryzae* (Figure 9), and to further increase *Bgh* penetration success on *mlo ror* double mutants (Figure 7), while this concentration was sufficient to overcome *Mla1*-specified (*R* gene mediated) resistance (Figure 8). These findings further reinforce the previous conclusion that the molecular pathways required for the various types of disease resistance are different [65] and that polyamines may affect these pathways in a differential manner.

Polyamines can bind to various types of cellular macromolecules, such as DNA, RNA, and proteins [39]. For example, polyamines were reported to function as modulators of a number of cation channels by directly plugging their pores. These comprise strong inward rectifying potassium channels as well as calcium-permeable glutamate receptor channels [66–68]. In addition, they block cGMP-gated channels in some human cell types via a complex mechanism [69,70]. One may thus hypothesize that exogenously applied polyamines could exert their effect via disrupting ion homeostasis of epidermal cells, leading thereby to a failure of defense. In this respect, interference with cGMP signaling could be a common denominator of the activity of ribonucleosides/nucleobases and their derivatives on the one hand and polyamines on the other hand. Plants cells contain physiologically relevant concentrations of cGMP, and there is strong experimental evidence for the existence of cGMP-activated ion channels in plants [35,71,72]. The levels of free polyamines were reported to increase several-fold at areas surrounding powdery mildew colonies (so-called green islands), whereas such a significant increase was not observed at areas showing senescence [73,74]. Polyamines may thus increase susceptibility by supporting the establishment and maintenance of green islands surrounding pathogen penetration sites, which could provide sustainable nutrition for the fungal pathogen.

We noted that some of the chemicals that are capable of partially suppressing *mlo* resistance in barley (BFA and 2,3,-dideoxyadenosine) were also effective in suppressing resistance in the *Arabidopsis mlo2 mlo6* double mutant (Supplementary Figures S3A and S4B). This finding is in line with the knowledge that *mlo* resistance in different plant species (monocots and dicots) requires similar molecular pathways and/or components [22,47]. However, some of the compounds (spermine) that are able to overcome *mlo* resistance partially in barley were not effective in case of the *Arabidopsis mlo2 mlo6* double mutant (Supplementary Figure S8). This discrepancy could be due to different physiological conditions in barley and *Arabidopsis* and/or based on different bioavailability of the compounds in the two species. Alternatively, this outcome could reflect differential requirements for *mlo*-mediated resistance in barley and *Arabidopsis*. It remains a task for the future to unravel the detailed inhibitory mode of action that ribonucleosides and their derivatives, nucleobases, as well as polyamines exert on *mlo* resistance in barley and *Arabidopsis*.

## Materials and methods

### Plant and fungal materials

The following barley (*H. vulgare*) lines were used for the present study: cultivar (cv.) Ingrid (wild-type; *Mlo* genotype), *mlo-*3 (near-isogenic line in cv. Ingrid; [8]), *mlo-*5 (near-isogenic line in cv. Ingrid; [8]), *mlo-*5 *ror1*-4 (near-isogenic line in cv. Ingrid; [14]), *mlo-*5 *ror2*-1 (near-isogenic line in cv. Ingrid; [14]) and P01 (near-isogenic line in cv. Pallas containing *R* gene *Mla1*; [75]). The *A. thaliana* lines used in the present study are accession Col-0 (wild-type) and the *mlo2*-5 *mlo6*-2 double mutant in the genetic background of Col-0 [22]. All barley seedlings were grown at 23°C and 16-h light/8-h darkness in a controlled environment. All *Arabidopsis* seedlings were grown at 22°C and 8-h light/16-h darkness in a controlled environment. *Bgh* isolate K1 was propagated on barley cv. Margret, and *G. orontii* was propagated on susceptible *Arabidopsis* Col-0 and mutant *eds1*-2 (in Col-0 background; [76]). The *M. oryzae* isolate TH6772 was kindly provided by the Institute of Biochemistry, Tamagawa University (Machida-shi, Tokyo, Japan). Maintenance of the fungus and inoculation methods were previously described [77].

### Chemical treatments

Chemical treatments were performed as follows: in the case of barley, the lower (abaxial) epidermis of detached first leaves (8 days old) was carefully peeled off using forceps. Specimens were then floated in Petri dishes on solutions containing the respective compounds (see Supplementary Tables S1 and S2), with the abaxial side in contact with solutions. Thereafter, samples were vacuum infiltrated for 20 min. Subsequently, the upper (adaxial) epidermis of the leaves was inoculated with *Bgh* (K1) conidiospores by flapping the inoculum over an inoculation box, ensuring an equal inoculation density. In the case of *Arabidopsis*, detached rosette leaves (4–5 weeks old) were placed into a 2-ml reaction tube containing solutions of the respective compounds. Petioles were immersed in the solutions and samples then vacuum infiltrated for 40 min. Leaves remained *in situ* for 1 h for resting; thereafter they were transferred on to 1% agar plates (containing 85 µM benzimidazole) and inoculated with *G. orontii* conidiospores by brushing inoculum on the adaxial side of the leaves. For both barley and *Arabidopsis*, at 48 hpi leaves were fixed in destaining solution (a 1:3 mixture of acetic acid and ethanol), and epiphytic fungal structures were stained with Coomassie Brilliant Blue R-250 (C.I. 42660, Carl Roth, Karlsruhe, Germany; 0.05% in 45% (v/v) methanol/10% (v/v) acetic acid) for microscopic analysis. Quantitative assessment of host cell entry was performed by light microscopy as the proportion of germinated conidia that succeeded in the formation of microcolonies. At least 100 interaction sites were evaluated for each leaf. In most cases, water or DMSO served as solvent for the various compounds. In some instances, other solvents (e.g. ethanol) were used, where appropriate (see Supplementary Tables S1 and S2).

### Trypan Blue staining for cell death assessment

Leaves of barley line P01 treated with either water (control) or 0.1 mM spermine were collected at 48 and 72 hpi and boiled for 8 min in fresh Trypan Blue working solution. The respective stock solution consists of 10 ml dl-lactic acid, 10 g phenol, 10 ml glycerol, 10 mg Trypan Blue (T0776, Sigma–Aldrich, Taufkirchen, Germany) and 10 ml H_2_O; the working solution was a 1:1 mixture of stock solution with 100% ethanol. Subsequently, leaves were transferred into chloral hydrate for destaining for 1–2 h, and then preserved in 70% glycerol. Epiphytic fungal structures were stained with Coomassie Brilliant Blue R-250 for microscopic analysis. Images were taken with a Keyence BZ-9000 digital microscope (Keyence, Osaka, Japan) using default settings.

### Extraction, derivatization, and HPLC analysis of polyamines

Detached leaves of both barley *Mlo* and *mlo* genotypes were inoculated with *Bgh* conidiospores, and leaves frozen in liquid nitrogen at the respective time points (0, 12, and 24 hpi). Polyamines were extracted and derivatized with dansyl chloride as described [78] with some modifications. Frozen leaf samples (~200 mg fresh weight) were ground in liquid nitrogen with mortar and pestle to fine powder. The homogenate was transferred to 15-ml tubes, suspended in 4% perchloric acid (1 ml/200 mg tissue FW) and shaken at 4°C for 1 h. Samples were centrifuged at 15000 rpm for 15 min, supernatants were collected and if necessary stored at –20°C before subsequent derivatization. Aliquots of extracts (200 µl) were transferred into screw-capped glass vials and the following reagents were added to each vial: 0.4 ml dansyl chloride (10 mg/ml of acetone), 0.2 ml of saturated sodium carbonate solution, and 10 µl of 1,7-diaminoheptan (internal standard; 0.2 mg/ml). Reaction mixtures were vortexed vigorously and subsequently incubated (continuously shaken) at 60°C in darkness for 1 h. After this time, 0.1 ml of proline (100 mg/ml) was added to each reaction mixture to remove the excess dansyl chloride. Samples were incubated at room temperature in darkness for 30 min and dansylated polyamines were extracted with 1 ml toluene. Toluene fractions were collected, diluted with 1 ml of hexane, and subjected to solid phase extraction on Discovery SPE DSC-Si columns (3 ml; 500 mg; Sigma–Aldrich). Columns were first washed with 2 ml toluene and 2 ml triethylamine in toluene (0.5:10 v/v). After sample loading columns were dried and dansylated polyamines were eluted with 2× 1 ml ethyl acetate, both ethyl acetate fractions were combined and evaporated in a SpeedVac vacuum concentrator to dry the residue. Pellets were redissolved in 200 µl 80% DMSO and obtained samples were analyzed on an Agilent (Palo Alto, CA) 1200 HPLC system equipped with a fluorescence detector. Derivatized polyamines were separated on a Synergi Polar-RP column (100×2.0 mm, 2.5 μm; Phenomenex, Torrance, CA) with water as solvent A and 98% acetonitrile as solvent B at a flow rate of 0.25 ml/min at 16°C (gradient of solvent A: 50% at 0 min, 50% at 2 min, 10% at 9 min, 0% at 16 min). Dansylated putrescine, spermidine, and spermine were detected by fluorescence (FLD detector; excitation 350 nm, emission 525 nm) based on the comparison of their retention times with those of respective standards derivatized according to the same protocol. Quantitative analyses were based on peak areas of identified dansylated polyamines normalized to the peak area of the internal standard. Polyamine concentrations were calculated by referring to the normalized peak areas measured during analysis of known amounts of respective dansylated polyamine standards.

### Statistical analysis

Statistical analysis via one-way ANOVA was performed with the XL toolbox integrated into Microsoft Excel. *Post-hoc* analysis was conducted with the Bonferroni–Holm method.

## Supporting information

**Table:** 

**Table:** 

## Abbreviations

BAX: BCL2-associated X protein
BFA: brefeldin A
*Bgh*: *Blumeria graminis* f. sp. *hordei*
cAMP: cyclic adenosine monophosphate
cv.: cultivar
DDA: 2′,3′-dideoxyadenosine
FW: fresh weight
hpi: h post inoculation
MiTMAB: myristyltrimethylammonium bromide
Mla1: *Mildew locus a 1*
Mlo: *Mildew locus o*
Ror: *required for mlo-specified resistance*
SNARE: soluble *N*-ethylmaleimide-sensitive factor attachment protein receptor
