# Supplementary figures and images for: Chemical suppressors of mlo-mediated powdery mildew resistance
Source: Biosci Rep. 2017 Dec 12;37(6):BSR20171389. doi: 10.1042/BSR20171389 (PMC5725617; doi:10.1042/BSR20171389)

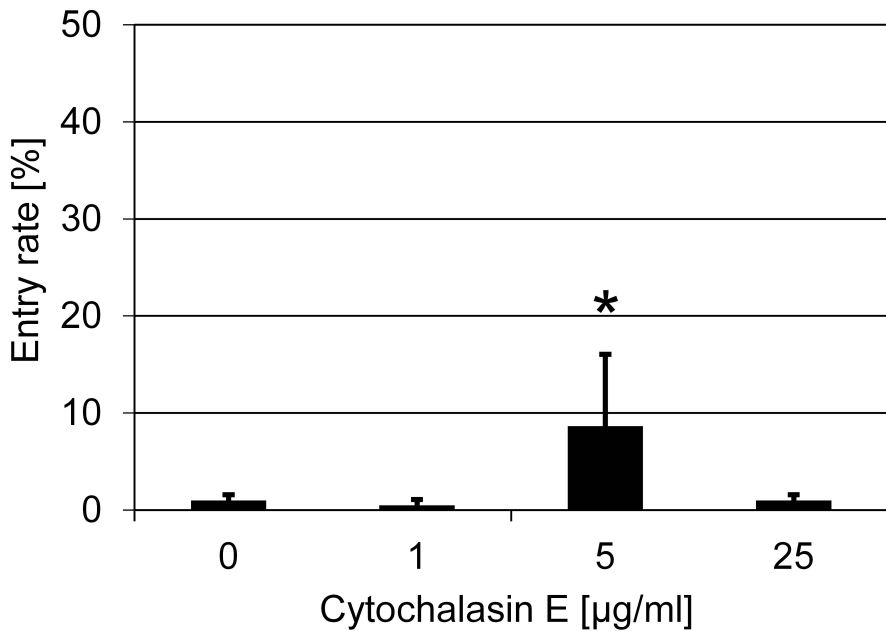

**A**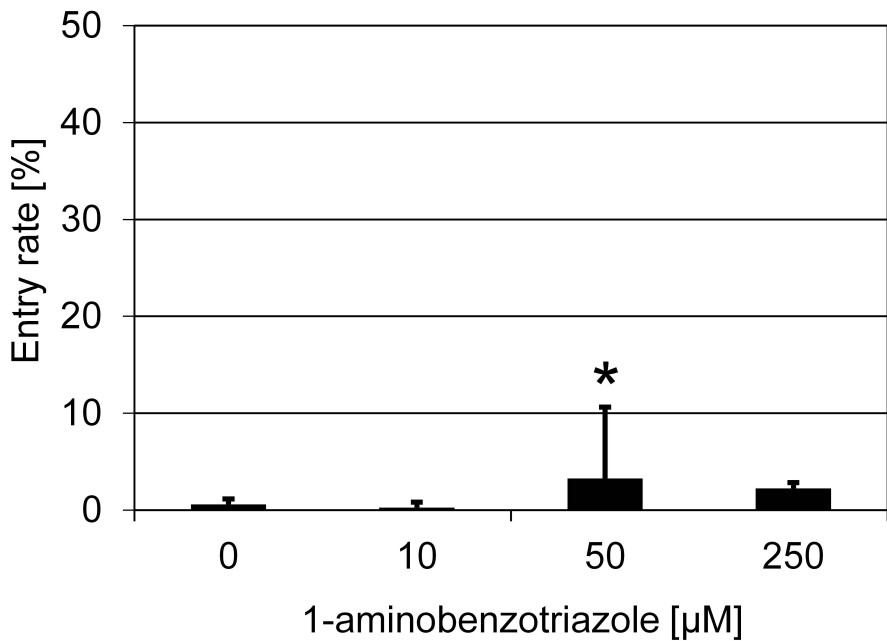**B**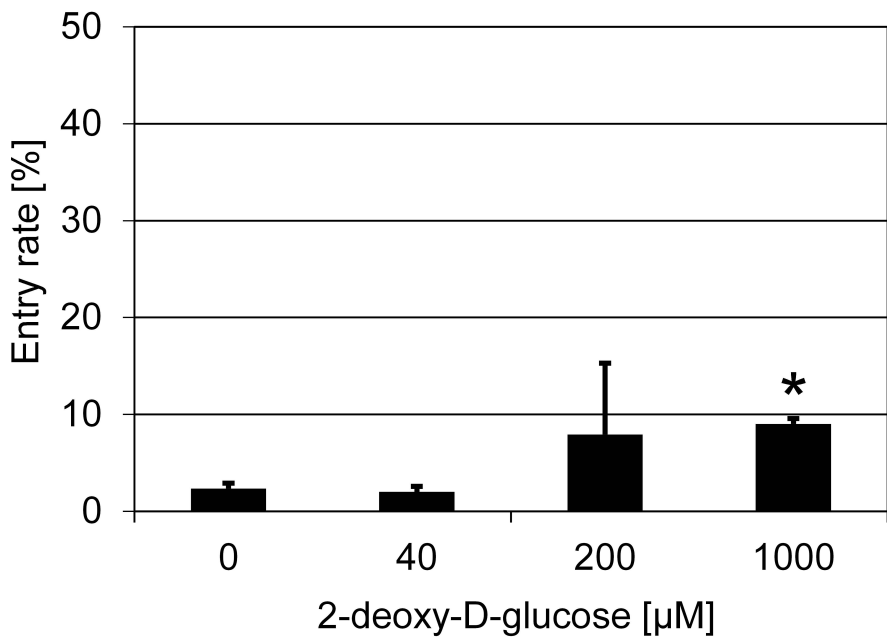

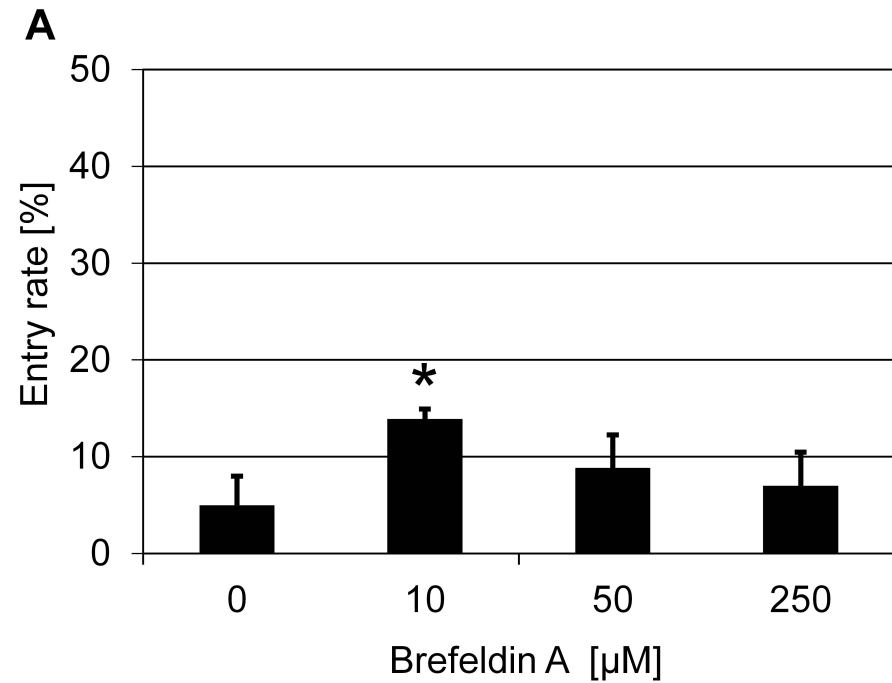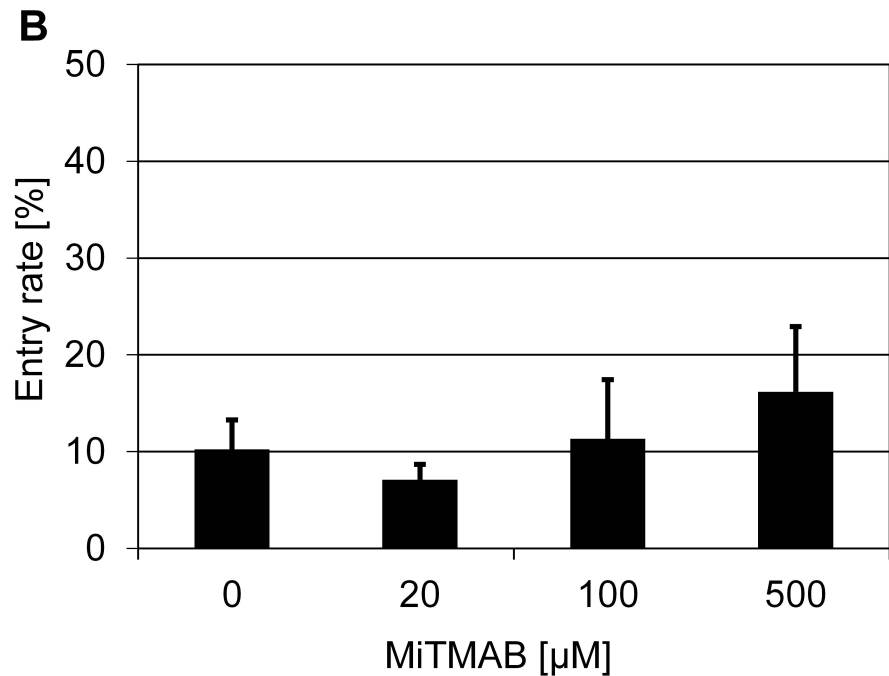

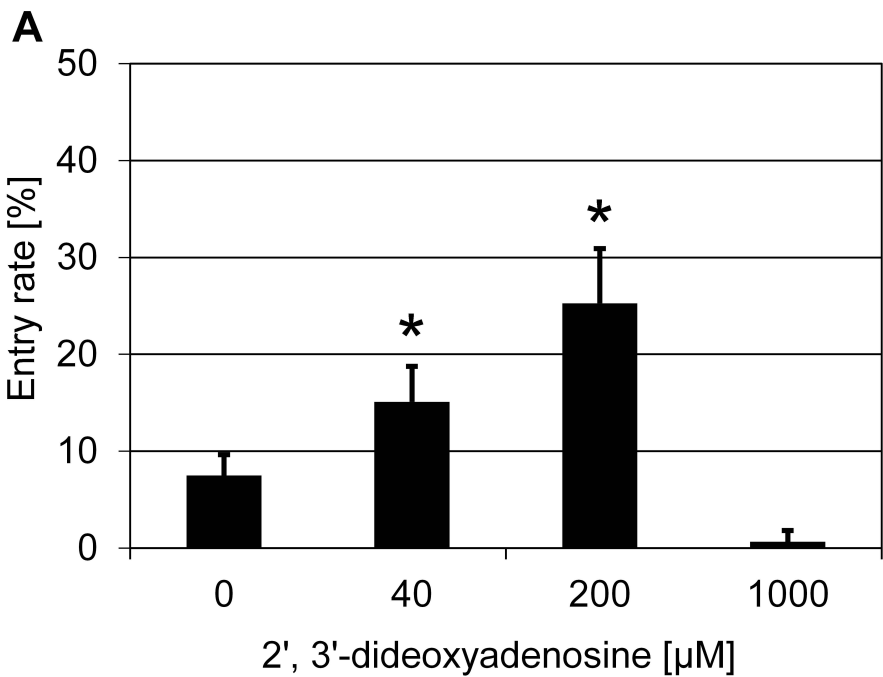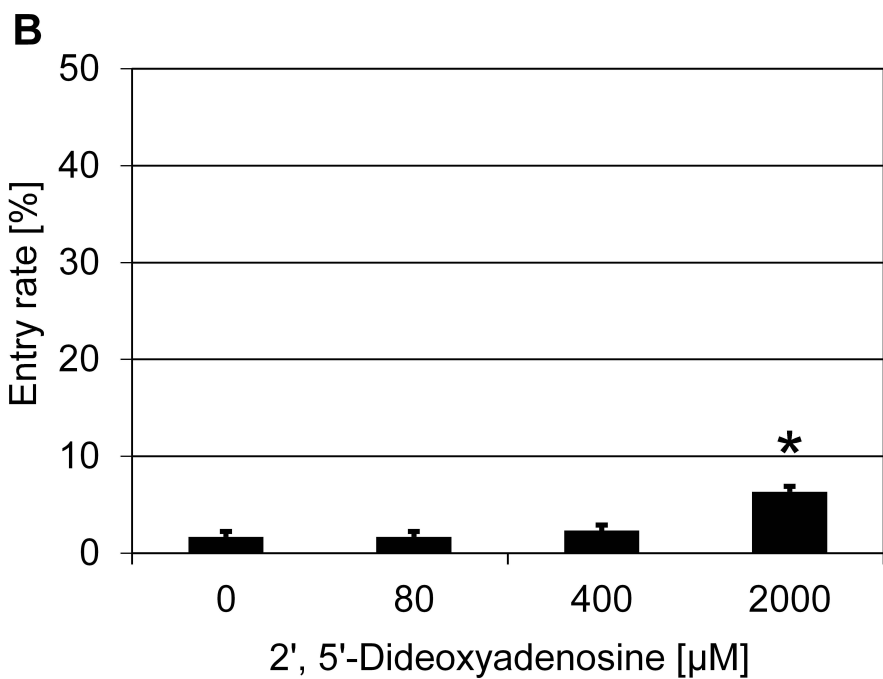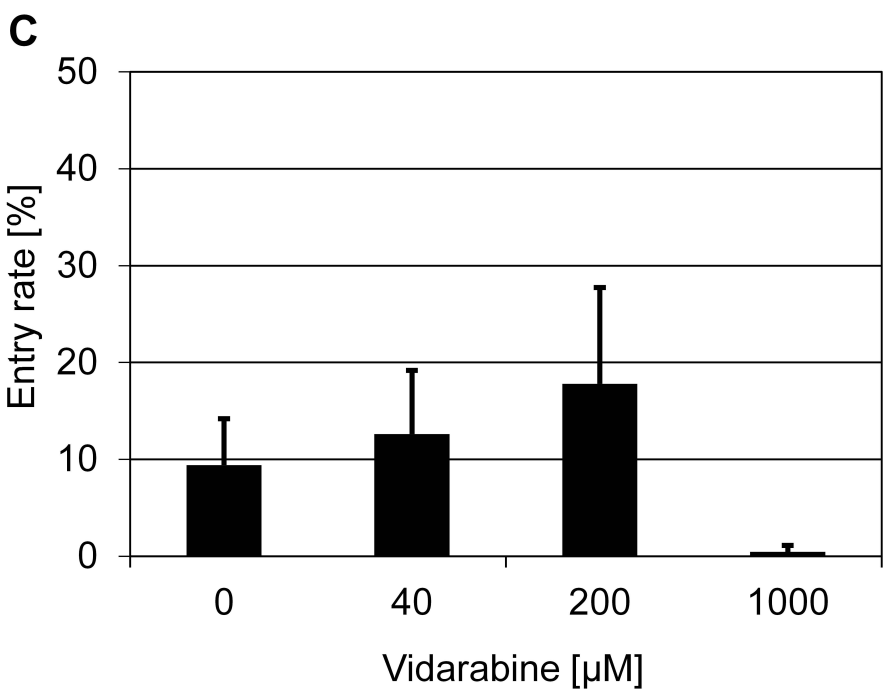

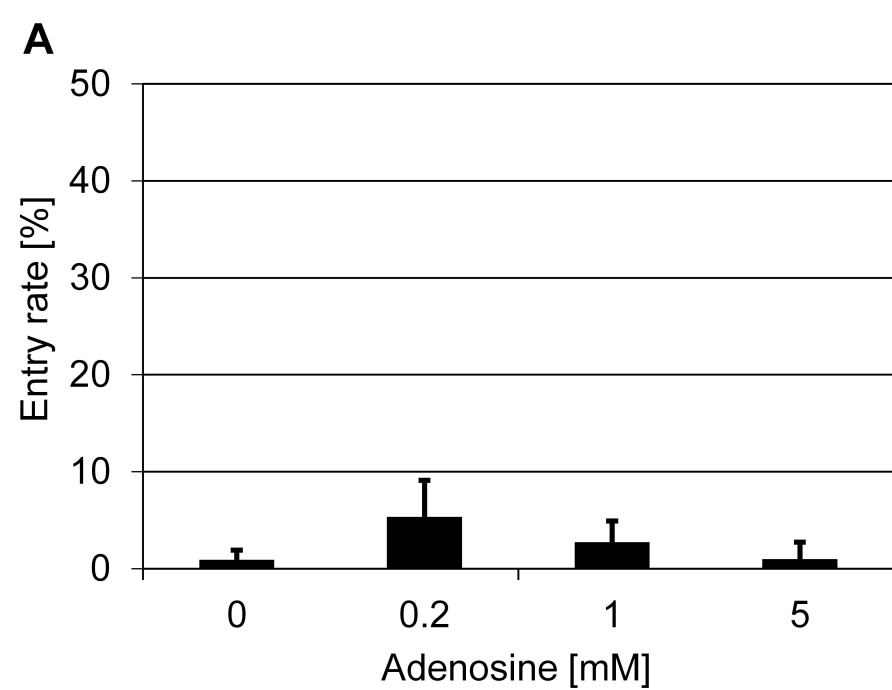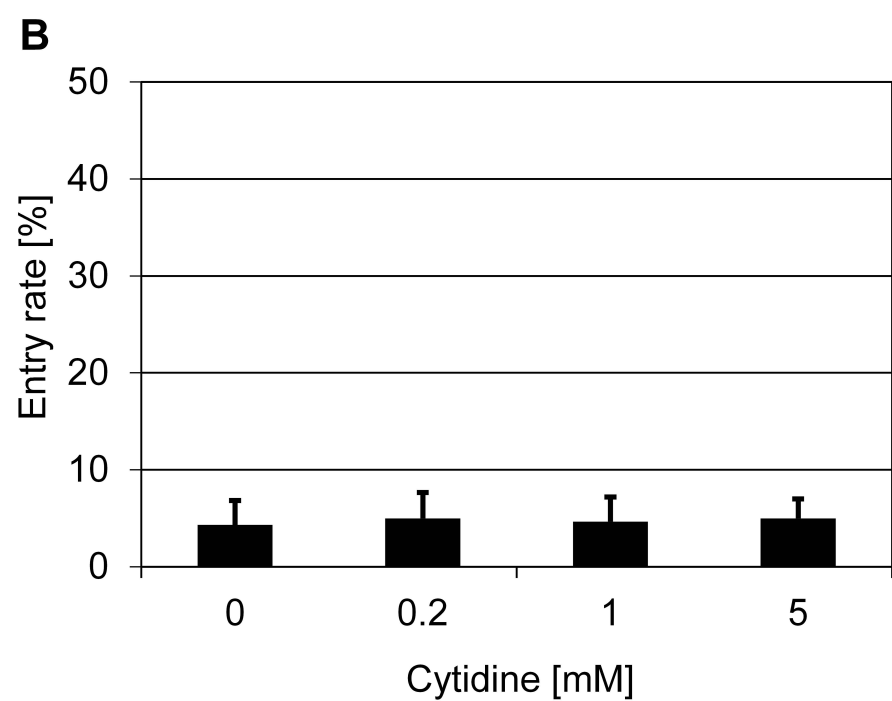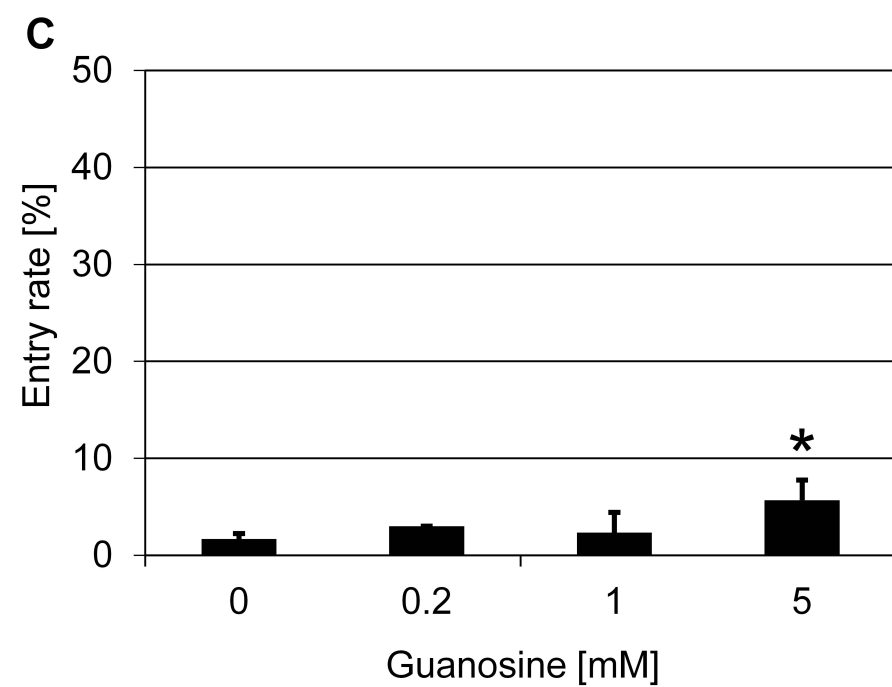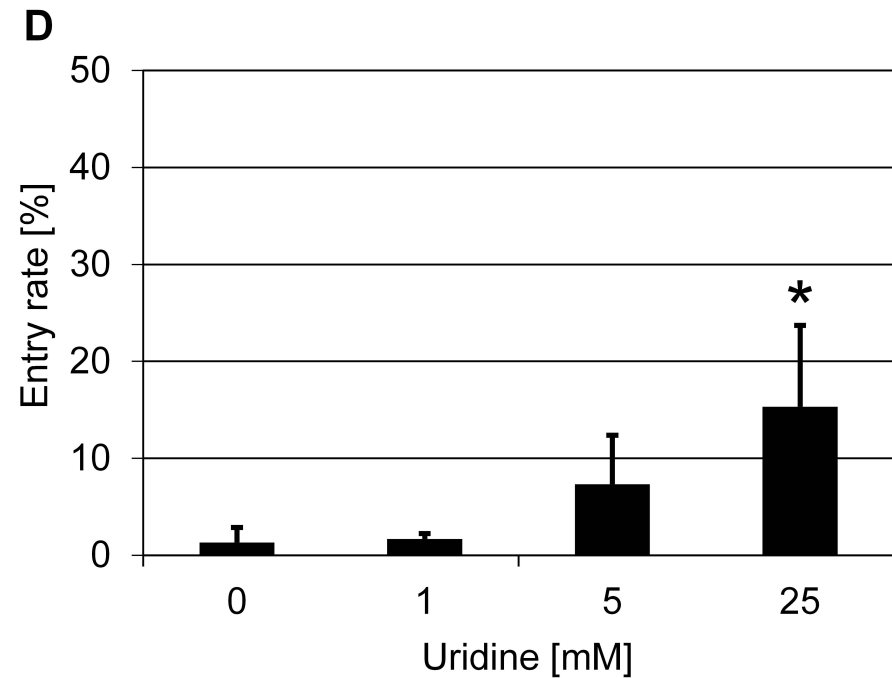

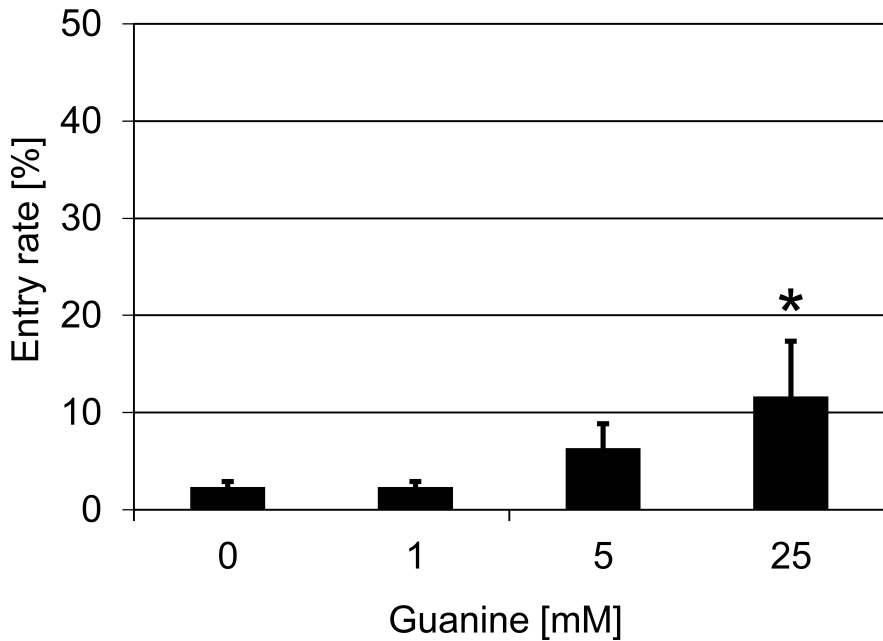

**A**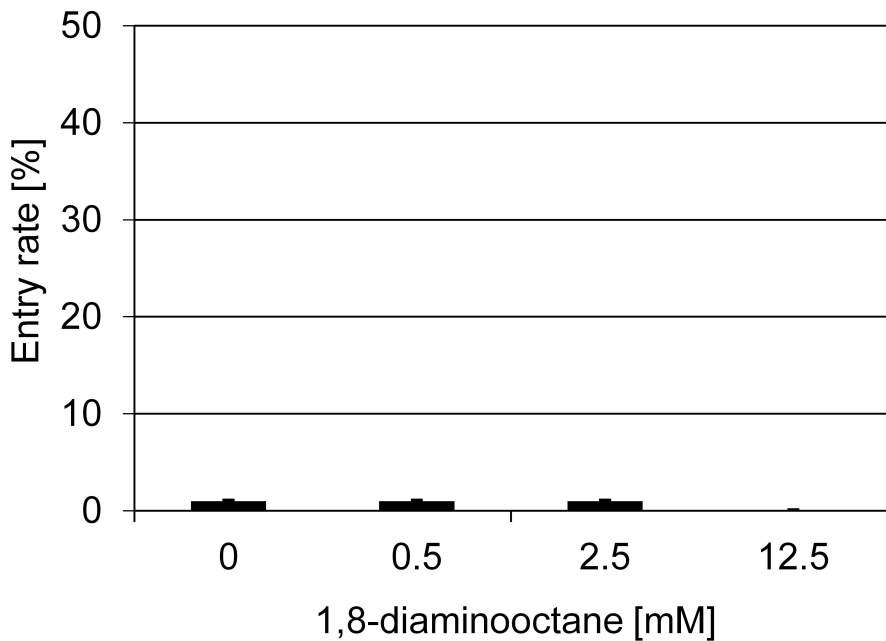**B**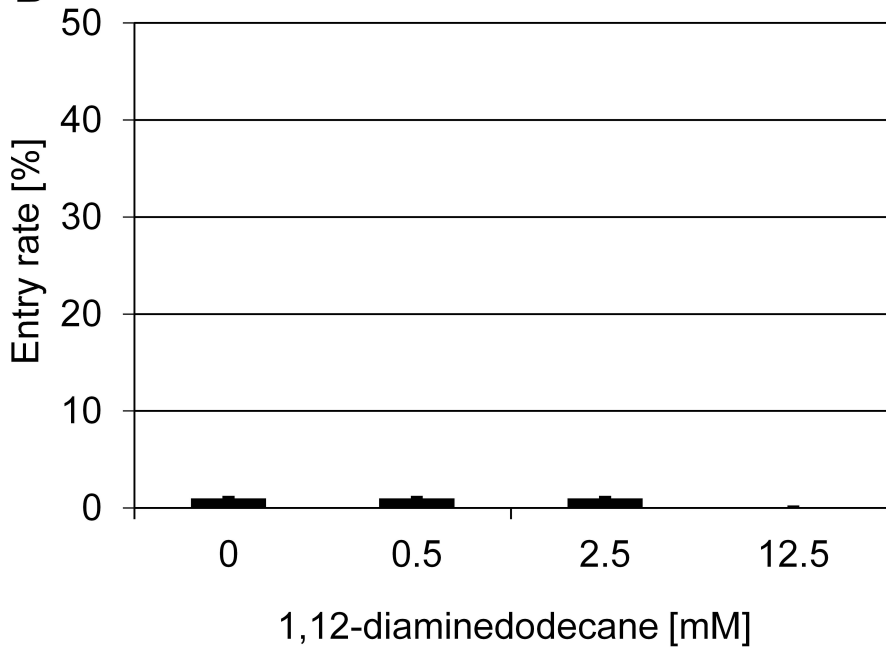

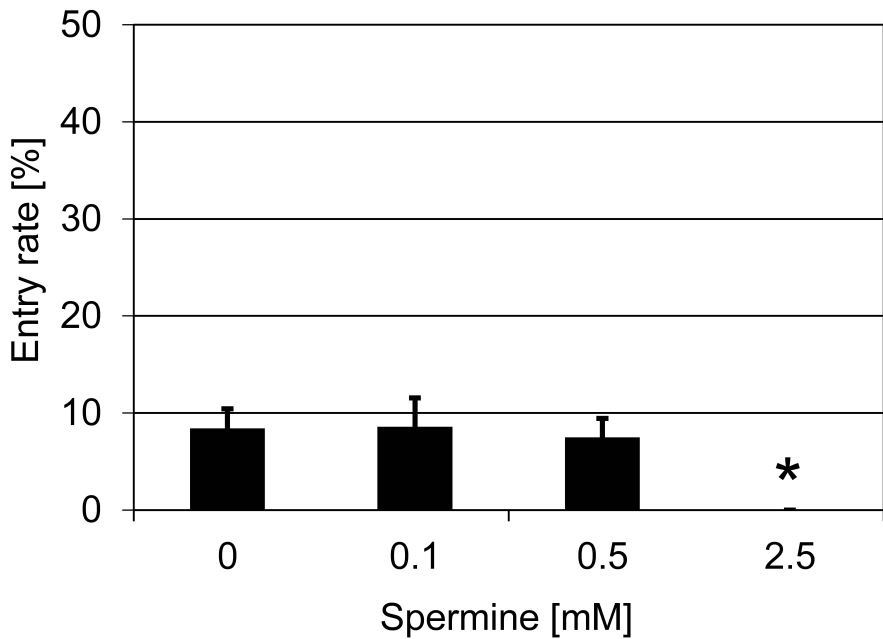

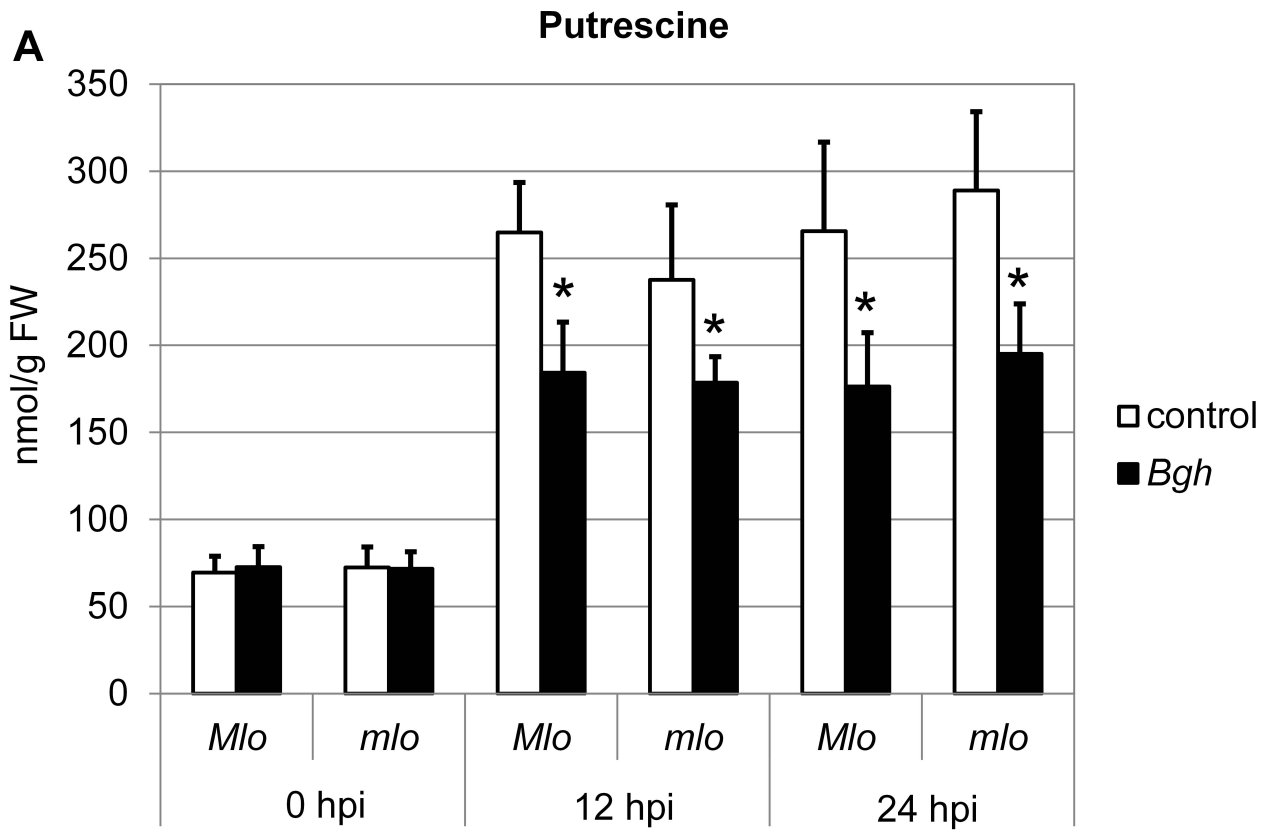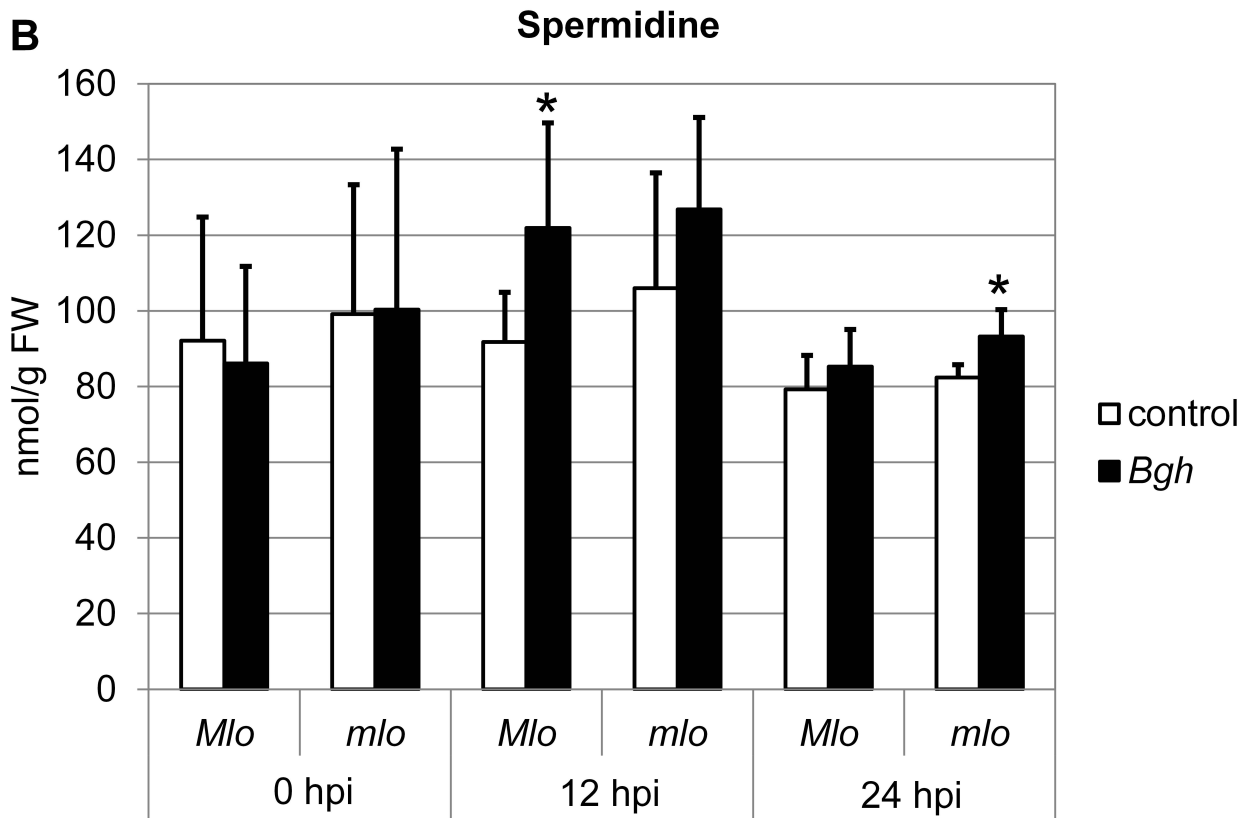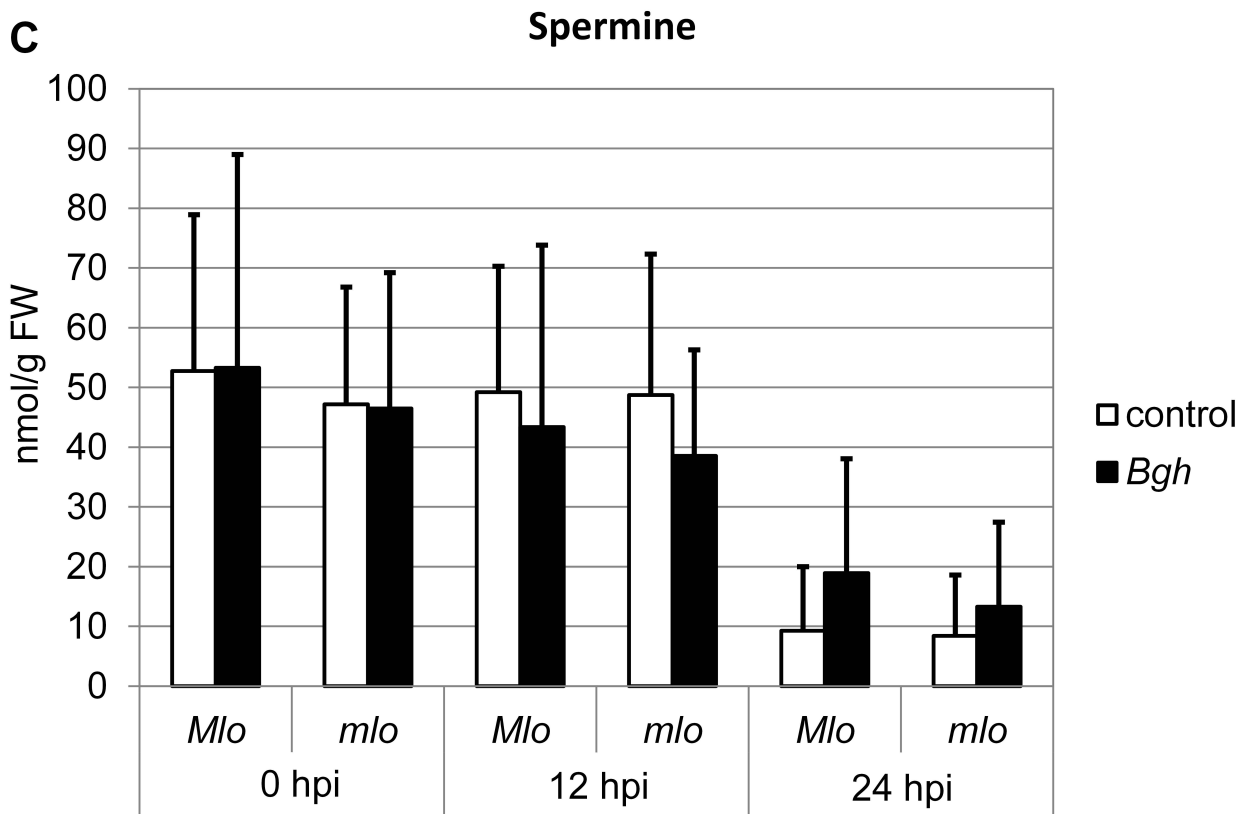

Supplement: Supplementary file 1 [file bsr20171389_Supp1.pdf]
